# Involvement of Chaperone Sigma1R in the Anxiolytic Effect of Fabomotizole

**DOI:** 10.3390/ijms22115455

**Published:** 2021-05-21

**Authors:** Mikhail V. Voronin, Yulia V. Vakhitova, Inna P. Tsypysheva, Dmitry O. Tsypyshev, Inna V. Rybina, Rustam D. Kurbanov, Elena V. Abramova, Sergei B. Seredenin

**Affiliations:** Department of Pharmacogenetics, Federal State Budgetary Institution “Research Zakusov Institute of Pharmacology”, Baltiyskaya Street 8, 125315 Moscow, Russia; tsypysheva.ip@gmail.com (I.P.T.); nomadussc@gmail.com (D.O.T.); zakusovpharm@mail.ru (I.V.R.); ramdacato@yandex.ru (R.D.K.); ryaskinv@mail.ru (E.V.A.)

**Keywords:** chaperone Sigma1R, fabomotizole, anxiolytic, elevated plus maze, (+)-pentazocine, NE-100, BD-1047, Sigma1R ligands, docking

## Abstract

Sigma-1 receptor (chaperone Sigma1R) is an intracellular protein with chaperone functions, which is expressed in various organs, including the brain. Sigma1R participates in the regulation of physiological mechanisms of anxiety (Su, T. P. et al., 2016) and reactions to emotional stress (Hayashi, T., 2015). In 2006, fabomotizole (ethoxy-2-[2-(morpholino)-ethylthio]benzimidazole dihydrochloride) was registered in Russia as an anxiolytic (Seredenin S. and Voronin M., 2009). The molecular targets of fabomotizole are Sigma1R, NRH: quinone reductase 2 (NQO2), and monoamine oxidase A (MAO-A) (Seredenin S. and Voronin M., 2009). The current study aimed to clarify the dependence of fabomotizole anxiolytic action on its interaction with Sigma1R and perform a docking analysis of fabomotizole interaction with Sigma1R. An elevated plus maze (EPM) test revealed that the anxiolytic-like effect of fabomotizole (2.5 mg/kg i.p.) administered to male BALB/c mice 30 min prior EPM exposition was blocked by Sigma1R antagonists BD-1047 (1.0 mg/kg i.p.) and NE-100 (1.0 mg/kg i.p.) pretreatment. Results of initial in silico study showed that fabomotizole locates in the active center of Sigma1R, reproducing the interactions with the site’s amino acids common for established Sigma1R ligands, with the ΔG_bind_ value closer to that of agonist (+)-pentazocine in the 6DK1 binding site.

## 1. Introduction

Epidemiological data indicates that global prevalence of anxiety disorders reaches 7.3% (95% CI 4.8–10.9%) [1,2,3]. For example, in the USA and China, the lifetime prevalence of generalized anxiety disorders is 6.2% and 4.7%, panic attacks 5.2% and 3.4%, and obsessive-compulsive disorders 2.7% and 3.2%, respectively [4,5]. According to E. Shirneshan et al., economic losses associated with anxiety disorders in the USA in 2013 reached 33.7 billion US dollars [6,7]. It is well established, that anxiety disorders are an etiopathogenetic factor for a number of somatic diseases, including acute cerebral circulation disorders [8], ischemic heart disease [9], neoplasms [10], and diabetes [11]. Therefore, an obvious necessity to prevent and treat anxiety disorders arises. Treatment of such disorders, both when they are initially diagnosed and in comorbid conditions, can be, when necessary, supported by medications.

Benzodiazepine tranquilizers were commonly used in the 1960s and 1970s of the last century for pharmacotherapy of anxiety disorders [12]. However, they still remain in medical practice [13]. Later, these tranquilizers were replaced by selective serotonin reuptake inhibitors (SSRIs) [2]. Use of benzodiazepine tranquilizers is limited due to their sedative and muscle relaxant effects as well as ataxia and impaired cognitive functions [14], which appear simultaneously with anxiolytic effects [15]. Benzodiazepines are known to cause addiction [16]. One of the SSRIs’ disadvantages is their relatively long latency period before the emergence of the anxiolytic effect [17]. It is noted that approximately 40% of patients are nonresponsive to SSRIs and serotonin-noradrenaline reuptake inhibitors (SNRIs) [18], anxiolytic effects of which range from mild to moderate (effect sizes 0.37–0.44) [2].

Thus, an acute problem of finding new anxiolytics with rapid effect onset, and lack of sedation and side effects, arises. Various approaches to the development of new anxiolytics regulating GABAergic, serotonergic (5-HT), glutamatergic, endocannabinoid, and neuropeptide systems are presented in a number of reviews and monographs [19,20,21,22].

Modern fundamental research makes it possible to highlight the chaperone Sigma1R as a promising target for anxiolytics development. Sigma1R is expressed in human [23] and rodent brain structures [24,25,26,27,28]. It has been shown that *Sigmar1^−/−^* mice exhibit an anxiety-like phenotype of behavior in the open field test (OF) and the elevated plus maze test (EPM) [29]. On the other hand, Sigma1R activation prevents the development of anxiety-like behavior [30,31].

Human and rodent Sigma1R proteins contain 223 amino acid residues (25 kDa), which are more than 90% identical. The chaperone has a unique amino acid sequence and is not homologous to known mammalian proteins [32,33]. A crystal structure has been determined for Sigma1R in which protein forms a homotrimer with one transmembrane domain and a binding site in each protomer [32,34,35,36]. Amino acid residues responsible for chemical binding have been identified, and conformational changes in Sigma1R caused by the prototype agonist (+)-pentazocine (PTZ) have been described [35]. Within cells, Sigma1R is predominantly localized in the cholesterol-rich region of endoplasmic reticulum (ER) membranes contact with mitochondria (MAM) [37,38,39,40], where it regulates functional activity of resident proteins through chaperone interactions [38,39]. Within lipid microdomains, the chaperone gains capabilities of redistribution within the ER and translocation to the region of plasma and nuclear membranes, both of which happen upon ligand activation or under conditions of cellular stress [41,42,43,44].

Sigma1R engages into protein–protein interactions, including engagement with proteins contributing to anxiolytic-like activity in the plasma membrane region. The regulatory effect of Sigma1R on the complex of cannabinoid and glutamate receptors with redox-regulated HINT1 protein (CB1R–HINT1–GluN1) has been established [39,45]. Along with anxiety-like behavior, *Cnr1^−/−^* and *Sigmar1^−/−^* mice are characterized by an increased level of GluN1, which bears the regulatory C1 cytosolic segment in the membranes of frontal cortex neurons [29,46]. Chaperone activity of Sigma1R towards ASICs channels causes their inhibition [47,48], which is considered to be one of the anxiolytic-like action mechanisms [49]. The mesolimbic dopamine system is known to play a role in the stress response formation [50] and pathogenesis of anxiety disorders [51]. Therefore, the Sigma1R chaperone can contribute to anxiolytic effect through the established interaction with D_1_, D_2_ receptors, DAT and regulation of these proteins’ activity [52,53,54,55].

The oxidative stress role in the pathogenesis of anxiety disorders has been shown [56]. According to T. Hayashi, psychological stress is associated with increased ROS production, cellular stress response triggering, and unfolded protein response (UPR) activation in the ER [57]. Chaperone Sigma1R plays an important role in protecting cells from oxidative stress and ER stress. Sigma1R activation attenuates ROS formation in vitro [58,59]. During agonist-dependent dissociation from the main chaperone ER BiP (GRP 78, HSPA5) [41,60], Sigma1R stabilizes the ER stress sensor IRE1, prolonging its dimerization, enhancing endonuclease activity and production of a functionally active transcription factor XBP1, which induces expression of neurotrophins, antioxidant defense proteins, and chaperones genes [61,62].

Thus, it is possible that compounds possessing agonist-like activity toward Sigma1R would be able to induce an anxiolytic effect by regulating functional activity of target proteins, inhibiting prooxidant mechanisms, and activating UPR adaptive response.

The results of the fundamental studies discussed above are consistent with the data obtained from fabomotizole (ethoxy-2-[2-(morpholino)-ethylthio]benzimidazole dihydrochloride) investigation. The compound was registered in the Russian Federation in 2006 as a selective anxiolytic [15,63]. Complementarily, in experimental models in vitro and in vivo, the drug also exhibited cytoprotective [64], neuroprotective [65,66,67,68,69,70,71,72,73], and antidepressant-like [74] effects, and reduced movement disorders caused by the Sigma1R antagonist haloperidol [75].

Radioligand assay conducted in vitro (Eurofins Cerep) established the affinity of fabomotizole (FAB) to chaperone Sigma1R (K_i_ = 5.9 µM), regulatory sites of NRH: quinone reductase 2 (NQO2, MT_3_ receptor K_i_  =  0.97 µM) and monoamine oxidase A (MAO-A K_i_  =  3.6 µM), which could be related to an anxiolytic effect [76]. Additionally, in ex vivo experiments, fabomotizole displaced [^3^H](+)-pentazocine from the binding sites in the P2 fraction of the brain homogenates of ICR (IC_50_ = 13.7 µM), C57Bl/6 (IC_50_ = 7.7 µM), and BALB/c mice (IC_50_ = 6.4 µM) [77,78]. IC_50_ values turned out to be close to those previously calculated for the human Jurkat T-cell line (IC_50_ = 7.1 µM) [76].

Several lines of evidence suggest that experimentally and clinically established anxiolytic properties of fabomotizole [15,63,79] may appear due to its interaction with the Sigma1R chaperone. In particular, the Hill coefficient of fabomotizole (nH = 0.9) corresponds to the one obtained for (+)-pentazocine (nH = 0.91) [35,76]. Fabomotizole is capable of displacing another radioligand with agonist activity to Sigma1R [G-^3^H]PRE-084 [78], which suggests that fabomotizole and Sigma1R agonists have similar locations in the chaperone binding site. It is important to notice that in HT-22 immortalized mouse hippocampal cells, fabomotizole (10 nM) caused translocation of Sigma1R toward cytoplasmic membrane 30 and 60 min after exposure [80], which is consistent with agonist effect on Sigma1R [41]. Involvement of Sigma1R in fabomotizole pharmacodynamics was shown in in vitro and in vivo experiments, where the drug’s effects were similar to those of PRE-084, which is considered to be a selective agonist of Sigma1R [64,71,73]. At the same time, selective Sigma1R antagonists abolished the cytoprotective and neuroprotective effects of fabomotizole in the experimental studies [64,65,66,67,68,69,71,73].

To specifically test the hypothesis that fabomotizole could function in a Sigma1R agonist-like manner and provide anxiolytic effects, we used an approach combining in silico methods and in vivo experiments [81]. Dependence of anxiolytic properties of fabomotizole on Sigma1R was examined in the elevated plus-maze with male BALB/c mice in the presence of known Sigma1R antagonists. We performed complementary in silico studies using a molecular docking procedure to antagonist NE-100 and agonist (+)-pentazocine binding sites, whose crystallographic structures (PDB IDs: 6DK0 and 6DK1) were extracted from RCSB PDB (https://www.rcsb.org (accessed on 18 May 2021)) to gain an insight into the basis of fabomotizole interaction with Sigma1R.

## 2. Results

### 2.1. Elevated Plus Maze Test

**Figure 1:**
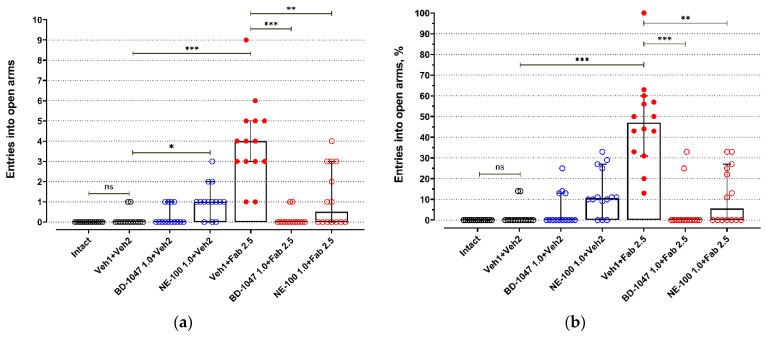
The influence of Sigma1R antagonists on fabomotizole anxiolytic dose effect expressed as the number of entries into the elevated plus-maze open arms. (**a**) Number of entries into the open arms (N open); (**b**) percentage of open arm entries (%N open). Experimental groups were divided by drug administration: intact BALB/c mice (Intact), vehicle 1 + vehicle 2 (Veh1 + Veh2), BD-1047 1.0 mg/kg + vehicle 2 (BD-1047 1.0 + Veh2), NE-100 1.0 mg/kg + vehicle 2 (NE-100 1.0 + Veh2), vehicle 1 + fabomotizole 2.5 mg/kg (Veh1 + Fab 2.5), BD-1047 1.0 mg/kg + fabomotizole 2.5 mg/kg (BD-1047 1.0 + Fab 2.5), NE-100 1.0 mg/kg + fabomotizole 2.5 mg/kg (NE-100 1.0 + Fab 2.5). Data are presented as median with 95% CI. Statistically significant differences according to the Kruskal–Wallis test and the post hoc Dunn’s multiple comparisons test: ns—not significant; * *p* < 0.05; ** *p* < 0.01; *** *p* < 0.001.

**Figure 2:**
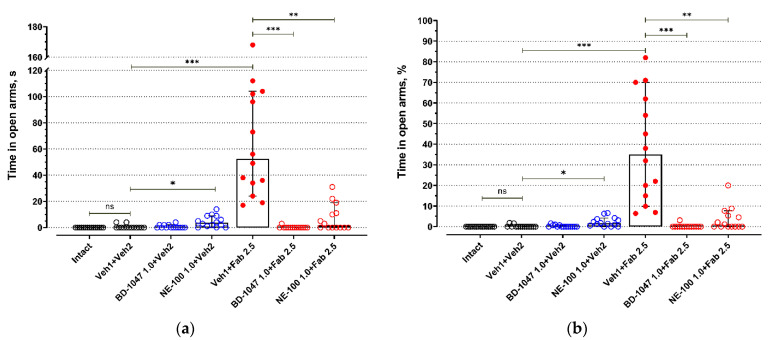
The influence of Sigma1R antagonists on fabomotizole anxiolytic dose effect expressed as time spent in the elevated plus-maze open arms. (**a**) time spent in open arms (T open in seconds); (**b**) percentage of time spent in open arms (%T open). Experimental groups were divided by drug administration: intact BALB/c mice (Intact), vehicle 1 + vehicle 2 (Veh1 + Veh2), BD-1047 1.0 mg/kg + vehicle 2 (BD-1047 1.0 + Veh2), NE-100 1.0 mg/kg + vehicle 2 (NE-100 1.0 + Veh2), vehicle 1 + fabomotizole 2.5 mg/kg (Veh1 + Fab 2.5), BD-1047 1.0 mg/kg + fabomotizole 2.5 mg/kg (BD-1047 1.0 + Fab 2.5), NE-100 1.0 mg/kg + fabomotizole 2.5 mg/kg (NE-100 1.0 + Fab 2.5). Data are presented as median with 95% CI. Statistically significant differences according to the Kruskal–Wallis test and the post hoc Dunn’s multiple comparisons test: ns—not significant; * *p* < 0.05; ** *p* < 0.01; *** *p* < 0.001.

BALB/c mice exhibited low activity in open arms of an elevated plus maze test (EPM) (Figure 1 and Figure 2, Table S1). Most BALB/c mice of the control groups (Intact, Veh1 + Veh2) do not enter open arms of the EPM (Figure 1, Table S1) or spend just a short time there (Figure 2, Table S1). Injections of control solutions did not affect the behavior of BALB/c mice in the EPM (Figure 1, Figure 2 and Figure S1). Fabomotizole administered at a 2.5 mg/kg dose 30 min prior to EPM exposition (Veh1 + Fab 2.5) led to a significantly higher number of entries into open arms and time spent in the open arms (*adj p* < 0.001) (Figure 1 and Figure 2, Table S1). Administration of BD-1047 (BD-1047 1.0 + Fab 2.5; *adj p* < 0.001) or NE-100 (NE-100 1.0 + Fab 2.5; *adj p* < 0.01) at a 1.0 mg/kg dose 30 min prior to fabomotizole prevented its effect and enhanced the anxiety-like behavior, reducing entries into open arms (Figure 1 and Figure 2, Table S1).

Administration of BD-1047, a Sigma1R antagonist, at a 1.0 mg/kg dose 60 min prior to EPM (30 min prior to vehicle 2; BD-1047 1.0 + Veh2) did not affect exploring activity of BALB/c mice in open arms (Figure 1 and Figure 2, Table S1). However, Sigma1R antagonist NE-100 administered at the same dose 60 min prior to EPM (30 min prior to vehicle 2; NE-100 1.0 + Veh2) moderately increased the number of entries into open arms (*adj p* = 0.032), but did not affect the number of entries expressed as a percentage of total entries into open and closed arms (Figure 1, Table S1). Administration of NE-100 also slightly increased the time spent in open arms expressed in seconds (*adj p* = 0.048) and percentage of total time spent in open and closed arms (*adj p* = 0.041) (Figure 2, Table S1).

Fabomotizole (Veh1 + Fab 2.5) moderately raised the number of total entries (*adj p* = 0.045) (Figure S1, Table S2). Under administration of Sigma1R antagonist BD-1047 (BD-1047 1.0 + Fab 2.5) 30 min prior to fabomotizole, a statistically significant decrease in the number of closed arms entries and the number of total entries occurred (Figure S1, Table S2). Less entries into closed arms were registered compared to the control group (Veh1 + Veh2; *adj p* < 0.014), the group with BD-1047 administration 60 min prior to exposition in EPM (BD-1047 1.0 + Veh2; *adj p* < 0.001), and the group with administration of NE-100 30 min prior to fabomotizole (NE-100 1.0 + Fab 2.5; *adj p* < 0.001) (Figure S1, Table S2). Total entries count decreased compared to the same groups and the group with control solution injection prior to fabomotizole (Veh1 + Fab 2.5; adj *p* < 0.001) (Figure S1, Table S2). Unlike NE-100, BD-1047 did not affect the number of entries into closed arms significantly compared to the fabomotizole group (Veh1 + Fab 2.5) (Figure S1, Table S2).

### 2.2. In Silico Docking Study

Crystal structure of Sigma1R revealed that protein exists as a trimer, and protomers are associated to form a flat triangle with the transmembrane region at the corners. Each monomer has a single N-terminal transmembrane domain and C-terminal region, containing a β-barrel cupin-like domain with a ligand-binding site, flanked by two hydrophobic α-helices [34]. The C-terminal region of the receptor faces the lumen of the endoplasmic reticulum, and the short sequence, preceding the transmembrane region, faces the cytoplasm. A flat hydrophobic surface, represented by two α-helices, likely faces the membrane and is embedded within the lipid bilayer [82].

Currently, Sigma1R structures in complex with compounds considered as antagonists (haloperidol (PDB ID: 6DJZ) [35], PD144418 (PDB ID: 5HK1) [34], NE-100 (PDB ID: 6DK0) [35]), agonist (+)-pentazocine (PDB ID: 6DK1) [35], and ambiguous ligand 4-IBP (PDB ID: 5HK2) [34] have been solved. Complementing earlier results [83], these data helped to identify the amino acid residues comprising the binding site of Sigma1R, which are involved in the binding of chemically divergent ligands. The Sigma1R binding pocket is located in the center of a β-barrel and is mostly lined with hydrophobic amino acids. However, Glu172 acidic residue was identified as essential for binding with chemically diverse ligands. In particular, highly conserved Glu172 generally forms hydrogen bonds with the protonated ligand’s amine group. Another acidic residue, Asp126, forms an intramolecular hydrogen bond with Glu172 and also plays a role in ligand binding. Additionally, hydrophobic Tyr103 contributes to hydrogen bonding with Glu172. It is currently believed that orientation of Glu172 is stabilized and fixed by interactions with Asp126 and Tyr103. Other amino acids that are involved in the ligand’s binding include hydrophobic Val84, Trp89, Met93, Leu95, Leu105, Phe107, Ile124, His154, Trp164, Leu182, which interact with the hydrophobic sites of the ligands, and Tyr103, which implicates in an aromatic stacking interaction in ligands [32,34,35,36,83,84].

To perform in silico modeling study the A chain of Sigma1R was used for further fabomotizole docking procedures. Furthermore, we used the reported 6DK1 and 6DK0 crystal structures bound to reference ligands (+)-pentazocine (selective agonist) and NE-100 (selective antagonist), respectively.

Despite the coincidence of the amino acid sequences (they are conservative), it should be taken into account that the binding sites of NE-100 and (+)-pentazocine (PDB IDs: 6DK0 and 6DK1) are not identical due to the fact that their geometric parameters were obtained as a result of co-crystallization of the same protein with reference ligands of different chemical structures. A preliminary analysis of the 6DK0 and 6DK1 binding sites showed that the criterion established by Schmidt [35], namely, that the ‘gap’ between the α4- and α5-helixes in the 6DK1 site associated with (+)-pentazocine is ‘slightly wider’ than in the structures associated with the known Sigma1R antagonists, are correctly accounted for in our docking study (see Figures S2–S4). It is logical to assume that the observed difference in the side chains geometry can modulate interaction of ligands with the receptor, which was confirmed by the results of the cross-docking procedure for reference ligands (+)-pentazocine and NE-100 and comparative docking of studied fabomotizole into 6DK1 and 6DK0 binding sites.

**Figure 3:**
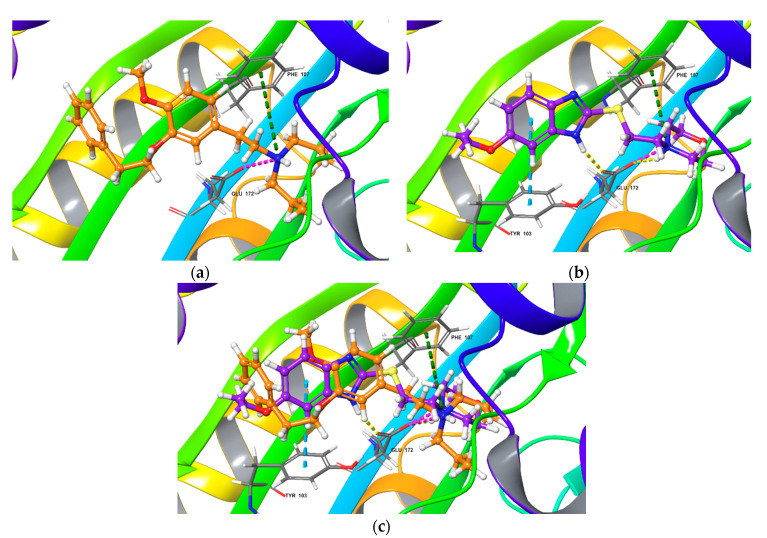
Top-scored docking poses for NE-100 and fabomotizole bound to Sigma1R binding site (PDB ID: 6DK0). (**a**) NE-100; (**b**) fabomotizole; (**c**) superposition of NE-100 and fabomotizole. Dotted green line—‘π-cationic’ interactions, dotted yellow lines—H-bonds, dotted purple lines—‘salt-bridges’, dotted blue lines—‘CH-π’ interaction.

**Figure 4:**
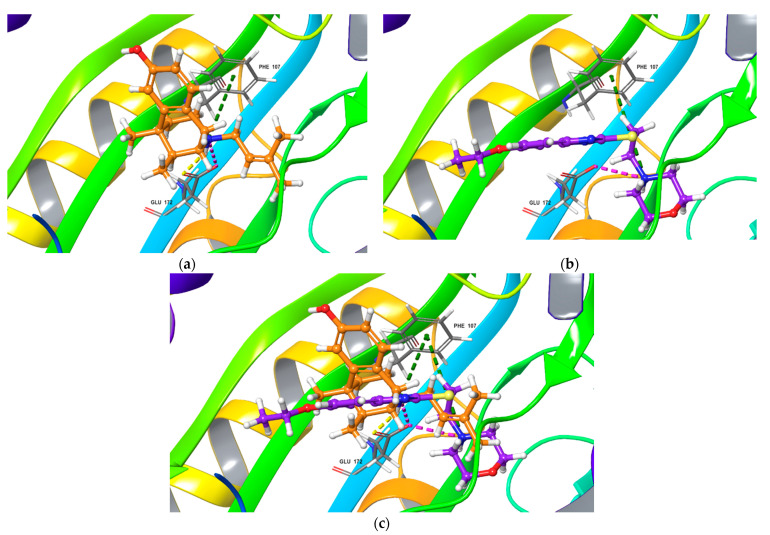
Top-scored docking poses for (+)-pentazocine and fabomotizole bound to Sigma1R binding site (PDB ID: 6DK1). (**a**) (+)-pentazocine; (**b**) fabomotizole; (**c**) superposition of (+)-pentazocine and fabomotizole. Dotted green line—‘π-cationic’ interactions, dotted yellow lines—H-bonds, dotted purple lines—‘salt-bridges’.

**Figure 5:**
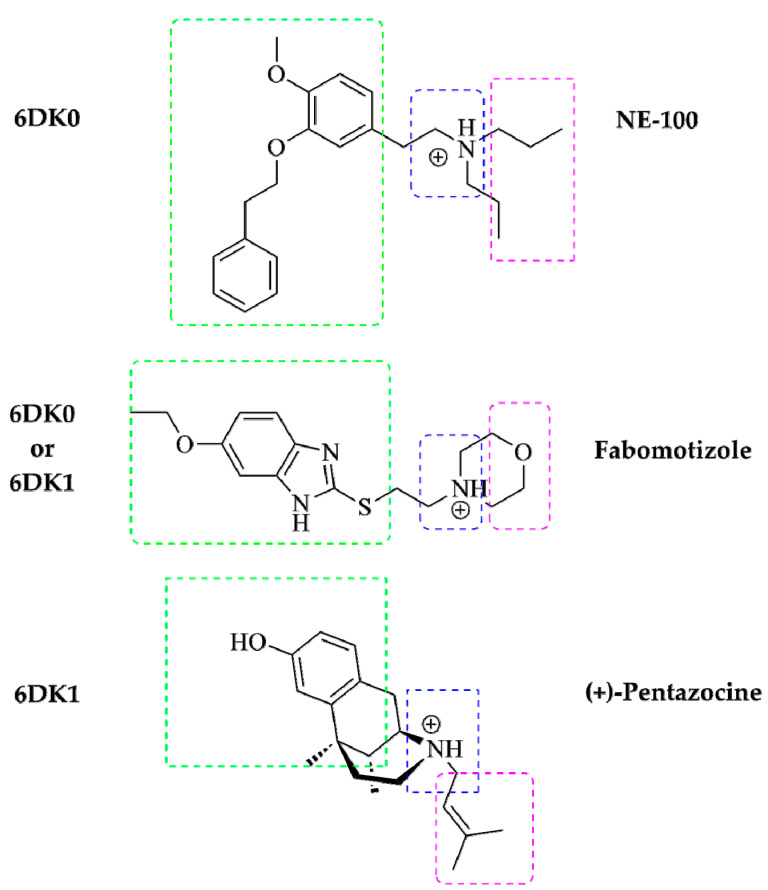
A schematic diagram of pharmacophore model for Sigma1R ligand based on R. Glennon et al. [87]. Shared pharmacophore features of NE-100, (+)-pentazocine and fabomotizole and their locations in the reference crystallographic structures of Sigma1R (PDB IDs: 6DK0 and 6DK1). Large hydrophobic region—green, small hydrophobic region—purple, ‘N-zone’—blue.

The results of computational experiments are summarized in Table 1 and presented in Figure 3, Figure 4 and Figure 5 and Supplementary Figures S5–S7. To determine the validity of the docking protocol, co-crystallized ligands, NE-100 or (+)-pentazocine, were re-docked into corresponding 6DK0 and 6DK1 sites of Sigma1R. The best ranked solutions of NE-100 and (+)-pentazocine exhibited a RMSD (root-mean-square deviation) value of 1.032 Å and 0.30 Å from the position of the appropriate co-crystallized ligand, respectively, indicating that the docking protocol was able to reproduce the binding mode of reference ligands (Table 1). Generally, RMSD values of less than 2.0 Å are considered to be indicative of the applied docking protocol accuracy [85].

The poses of antagonist NE-100 and fabomotizole at the Sigma1R binding site 6DK0 and interacting amino acid residues are represented in Figure 3. Docked NE-100 structure (Figure 3a and Figure S5a) was similar to that determined by Schmidt et al. [35]. Nitrogen atoms of NE-100 and fabomotizole interact with two amino acids—Glu172 (a salt bridge) and Phe107 (π-cation, see Figure 3). In addition, one of the nitrogen atoms of the benzimidazole part of the fabomotizole molecule binds with Glu172 via H-bond, and CH-π stacking interaction of this fragment with Tyr103 takes place (Figure 3b). It is worth noting that the benzimidazole part of fabomotizole is located in the large hydrophobic region of Sigma1R binding site, indicating the similarity of fabomotizole and NE-100 docking poses, because substituted aromatic fragment of NE-100 also occupies this pocket (Figure 5). However, their calculated values of ΔG_bind_ (Table 1) are markedly different (ΔG_bind NE-100_ = −74.76 kcal/mol; ΔG_bind FAB_ = −61.27 kcal/mol). The superposition of NE-100 and fabomotizole in the 6DK0 site, as well as the modes in which they interact with key amino acid residues, are shown in Figure 3c.

Next step of our study aimed to compare interaction features of agonist (+)-pentazocine and fabomotizole with the Sigma1R binding site (PDB ID: 6DK1). The calculated parameters obtained as a result of molecular docking procedure are summarized in Table 1. The found mode of interactions of (+)-pentazocine and tested fabomotizole as well as their superposition in the 6DK1 binding site are shown in Figure 4.

Analyzing obtained results, our first note refers to the fact that docked (+)-pentazocine was comparable with that determined by Schmidt et al. [35]. (+)-Pentazocine tertiary amino group forms a salt bridge and an H-bond with Glu172. π -Cationic interaction with Phe107 was also determined (Figure 4a). Nitrogen atom of morpholine moiety of fabomotizole molecule interacts with Glu172 and Phe107 via salt bridge and π-cation formation (Figure 4b). The formation of an H-bond between the nitrogen atom of the benzimidazole part of fabomotizole and Glu172 is detected once more (as in the case of its docking into the NE-100 binding site), though a difference between these two docking results such as an absent CH-π interaction with Tyr103 was observed (compare Figure 3b and Figure 4b). Most importantly, sufficiently close values of the ΔG_bind_ calculated for the ‘ligand-protein’ complex formation (Table 1) were found in 6DK1 crystallographic data; values of ΔG_bind PTZ_ is −60.50 kcal/mol and ΔG_bind FAB_ = −56.65 kcal/mol.

The major pharmacophore features of typical Sigma1R’s ligands are determined and described in the literature. It is evaluated, that electrostatic interactions between ligand’s nitrogen atoms and the side chain of Glu172 carboxylate are crucial for Sigma 1R ligands, so, a presence of a charged nitrogen (as a secondary or tertiary amine group) in the ligand’s molecule is a necessity [34,35,36]. Additionally, the ligand’s molecule can bear a noticeable hydrophobic part in its structure (most often these are aromatic, heteroaromatic rings, alkyl chains, or hydrocarbon frameworks): it is necessary for the location of these large hydrophobic moieties to be near the Val84, Met93, Leu95, Leu105, Tyr206, Ile178, Leu182, and Tyr103 residues (primary hydrophobic site of Sigma1R, see Figures S4 and S5). In turn, their smaller hydrophobic groups (most often, these are various substituents bonded to amine nitrogen) must be located near the Phe107, Trp164, His154, and Ile124 residues (these residues are included in the secondary hydrophobic site, see Figures S8 and S9). In general, Sigma1R ligand molecule includes: a large and small hydrophobic parts connected by a spacer (its length and nature may be different), and at least one amino group that is located somewhere in between these parts or near one of them [86]. It should be noted that structures of NE-100, (+)-pentazocine and fabomotizole molecules correspond to the abovementioned pharmacophore characteristics of Sigma1R ligands (summarized in Figure 5).

Presence of similar pharmacophore features of these three molecules leads to the fact that, according to the results of docking (see Table 1), NE-100, (+)-pentazocine, and fabomotizole interact with amino acid residues in 6DK0 and 6DK1 crystallographic structures of Sigma1R in almost the same way. Altogether, fabomotizole, docked into the (+)-pentazocine—or NE-100-bound Sigma1R structures, generally matches well with the binding mode of corresponding solved Sigma1R/ligand structures [35]. In all cases (see Table 1, Figure 3 and Figure 4) fabomotizole, (+)-pentazocine and NE-100 are identically oriented in the Sigma1R active site. Figure 5 simplistically represents such locations of the fabomotizole in the regions of the Sigma1R binding site, limited by the volumes of reference ligands, extracted from the crystallographic database (PDB IDs: 6DK0, 6DK1). Additionally, according to calculations, the benzimidazole part of fabomotizole lies inside a large hydrophobic pocket (green) as well as substituted aromatic part of NE-100 molecule or azabicyclononane framework annelated with hydroxybenzene of the (+)-pentazocine (Figure 5).

## 3. Discussion

EPM is a generally accepted validated test for modeling anxiety-like behavior in mice or rats as well as preclinical studies of anxiolytic compounds. The test is based on the behavioral traits of rodents, which tend to explore mainly closed areas, avoiding open ones [88]. For detailed information on the test, see [89,90]. Anxiety-like behavioral phenotype characteristics of BALB/c mice, such as avoidance of open arms in the EPM test, was confirmed in the conducted experiments [15,91]. Anxiolytic-like action of fabomotizole was reproduced, expressed as an increase in the number of entries and time spent by mice in open arms, while leaving closed arm entries unchanged [15,79]. Fabomotizole anxiolytic dose (2.5 mg/kg i.p.) [15] effects proved to be similar to those of opipramol, a tricyclic compound, which, while possessing properties of a Sigma1R agonist [92,93], is unable to inhibit reuptake of serotonin, norepinephrine, or dopamine (IC_50_ > 10,000 nM) [92,94]. Opipramol administered 1 h before testing showed anxiolytic-like properties under EPM (1 and 2.5 mg/kg p.o.) and social exploration test (0.01–10 mg/kg p.o.) in experiments on rats. The efficacy of opipramol in mice was demonstrated in the half-elevated platform test and stress-induced hyperthermia test (3 mg/kg p.o.) [94].

Achieved results demonstrate the anxiolytic-like effect of fabomotizole and its elimination by the Sigma1R antagonists BD-1047 (1 mg/kg i.p.) and NE-100 (1 mg/kg i.p.). Results are consistent with the data on the activity of compounds bearing Sigma1R agonist properties in other models of anxiety disorders. The conditioned fear stress is seen as an experimental model of posttraumatic stress disorder (PTSD) and is used for preclinical studies of anxiolytics [95]. Sigma1R agonists (+)-SKF 10.047 (5–6 mg/kg s.c.) and dextromethorphan (30–40 mg/kg s.c.), after a single injection to male ddY mice or male Kbl Wistar rats 15 or 20 min before the conditioned fear stress increased the mobility of shocked animals in a dose-dependent manner [52,96,97,98]. Igmesine (JO-1784), a selective Sigma1R agonist administered at a dose of 30 mg/kg i.p. 30 min before the motility measurement had a similar effect on shocked Wistar rats [99]. Preliminary administration of Sigma1R antagonists BMY-14802 (10 mg/kg s.c.) and NE-100 (5 mg/kg i.p.) 30 and 45 min before the test eliminated effects of both (+)-SKF 10.047 and dextromethorphan [97,100]. In rat experiments BMY-14802 (1 mg/kg s.c.) abolished the negative effects of igmesine (0.1 µg/kg i.c.v.) and neuropeptide Y (NPY) (0.15 µg/kg i.c.v.) on the conditioned fear stress induced colonic hyperkinesia [101]. At the same time, agonists (+)-pentazocine (16 and 32 mg/kg s.c.) and DTG (8 mg/kg i.p.) administered no later than 30 min before the experiment did not affect the mobility of shocked mice or rats in the conditioned fear stress test [52,96,97]. Authors associate differences in agonists action under conditioned fear stress model with different populations of Sigma1R that are being interacted with [52,97]. Under conditioned fear stress, the original Sigma1R ligand finazine (25 mg/kg i.p.) administered 30 min before the test reduced freezing time in C57Bl/6 mice 24 h after they received a series of mild foot shocks. The compound had a similar effect on Danio rerio, causing a dose-dependent switch from freezing to escape behavior in the presence of strobe light [102].

In addition to synthetic Sigma1R agonists, endogenous neurosteroids with agonist properties are also capable of activating animal behavior in the conditioned fear stress test [98]. Thus, a 50 mg/kg s.c. injection of dehydroepiandrosterone sulfate (DHEAS) or pregnenolone sulfate (PREGS) 30 min before the test attenuated the conditioned fear stress in mice. Progesterone (PROG, 10–50 mg/kg s.c.), possessing Sigma1R antagonist properties, did not affect motor activity of male ddY mice in the test. However, a 5 mg/kg i.p. PROG administration 15 min prior to DHEAS or PREGS eliminated their effect on the motility of shocked mice, and a 10 mg/kg i.p. dose blocked the action of agonist (+)-SKF 10.047, which has higher affinity for Sigma1R [98]. PROG effect appeared to be similar to NE-100 selective antagonist (5 mg/kg i.p.) [97,98]. The action mechanism of a single Sigma1R agonists administration on the conditioned fear stress test is discussed by the authors from the standpoint of their effect on mesolimbic dopaminergic systems, demonstrated experimentally [52,98,100], which is consistent with the Sigma1R chaperone function activation in relation to dopamine receptors and DAT [53,54,55].

In another PTSD experimental model, Sigma1R agonists exhibit anxiolytic-like activity even after a short course of administration, e.g., PRE-084 (0.6 mg/kg i.p.) administered to Sprague Dawley rats daily for seven consecutive days after the single-prolonged stress procedure increased the distance and time spent in the center of OF. EPM testing 1 h after OF also revealed a decrease in anxiety-like behavior (more time spent in open arms and higher percentage of open arm entries) in animals receiving a PRE-084 course [30,31]. The authors provided experimental data demonstrating the involvement of BDNF in the pharmacodynamics of PRE-084 [30,31], which is consistent with our data on fabomotizole ability to restore the level of BDNF in the BALB/c mice hippocampus following stress exposure in OF under bright lighting conditions after a single injection [103]. 

Sigma1R agonists affected ICR mice obsessive-compulsive behavior modeled in the marble-burying test [104,105]. Single injection of Sigma1R agonists (+)-SKF 10.047 (10 mg/kg i.p.) or PRE-084 (60 mg/kg i.p.) 30 min prior to the test significantly reduced the number of buried marbles, without affecting locomotor activity. When administered 60 min before the test, ISSRs fluvoxamine (30 mg/kg p.o.), which has the properties of a Sigma1R agonist showed analogous action [106], similar to paroxetine which has a higher affinity for serotonin and norepinephrine transporters [107], but significantly less affinity for Sigma1R in comparison with fluvoxamine [104,108,109]. It is worth noting that selective Sigma1R antagonists BD-1047 (3 mg/kg i.p.) and BD-1063 (1 mg/kg i.p.) administered 30 min before SSRIs interfered with fluvoxamine effect, leaving paroxetine effect unchanged [104]. Marble-burying test results indicate the contribution of Sigma1R activation to the reduction of anxiety-like and compulsive-like behaviors.

Contribution of Sigma1R activation to alleviation of the compulsive disorder induced by social stress in male Sprague Dawley rats was shown using NE-100 antagonist [110]. Dehydroepiandrosterone (DHEA, 15 mg/kg s.c.), having Sigma1R agonist properties [111,112], when administered via a 5 days course, increased the cumulative percentages of males, showing intromission and ejaculation on one hand and reduced latencies of intromission and ejaculation on the other. A 3 mg/kg i.p. dose of NE-100 antagonist interfered with the DHEA action [110].

As shown in this work, Sigma1R antagonists BD-1047 and NE-100 prevented anxiolytic-like effect of fabomotizole in EPM while specific features of their action were recorded. The NE-100 compound produced a slight, though statistically significant, increase in the number of open arms entries and time spent there. Along with eliminating the anxiolytic-like effect of fabomotizole BD-1047 also reduced closed arm entries and total number of entries compared to the control groups. Additional research is required for elucidation of reasons behind unequal effects of used Sigma1R antagonists.

Along with the data on anxiolytic effect of Sigma1R agonists, some studies do not support the discussed mechanism, e.g., in the works of J.F. Navarro et al., Sigma1R agonist (+)-SKF 10.047 (4 and 8 mg/kg i.p.) administered to outbred OF1 mice 30 min before EPM testing exhibited anxiogenic-like effects [113]. It cannot be ruled out that this effect of (+)-SKF 10.047, on one hand, may be associated with antagonistic effects of the compound on phencyclidine (PCP) dependent sites of GluN (NMDA) receptors [114,115]. PCP is able to induce anxiogenic-like action in rats [116]. On the other hand, differences in the effects mediated by pharmacological regulation of Sigma1R may depend on the level of endogenous ligands (neurosteroids) [117], selected doses of compounds [118], behavioral phenotypes in the EPM [119], and features of anxiolytics effect [120] on experimental animals.

To evaluate the features of fabomotizole interaction with Sigma1R, an initial docking analysis was performed, considering the data on the dependence of anxiolytic-like action of fabomotizole on Sigma1R obtained in our study and taking into account that not all compounds considered as agonists of Sigma1R possess anxiolytic properties. 

Consistent with results obtained in silico, fabomotizole shares some key features, common to structurally divergent Sigma1R ligands [34,35,87]: it forms an H-bond and a salt bridge with Glu172, and a π-cationic interaction with Phe107, indicating that the mode of fabomotizole interaction with Sigma1R is similar to that observed for known Sigma1R ligands. However, fabomotizole differs from antagonist NE-100 by additional H-bonds with Glu172, Trp164 and CH-π interaction with Tyr103, discovered by docking analysis. It should be noted, that patterns of interaction mode determined for fabomotizole and (+)-pentazocine are closer, except for the H-bond formation (this bond is formed by nitrogen atom of benzimidazole fragment of fabomotizole molecule and carboxyl group of Glu172 (see Table 1 and Figure 4a). Most likely, this finding can be attributed to structural features of reference (+)-pentazocine (it is a polycyclic ‘cage-type’ molecule) and fabomotizole (its benzimidazole part is conjugated with morpholine by means of a thio ethyl spacer). Indeed, unlike the (+)-pentazocin’s tertiary nitrogen atom, which is rigidly fixed in its ‘cage-type’ structure, the morpholine fragment of fabomotizole rotates freely due to the mobile thio ethyl spacer. This, possibly, helps fabomotizol to more accurately reproduce the ‘pentazocin-like’ interaction mode with 6DK1 binding site of Sigma1R, as opposed to the antagonist NE-100 6DK0 site.

At the same time, it is necessary to pay attention to the fact that the ΔG_bind_ (MM_GBSA) values calculated for fabomotizole (Table 1) in the antagonist 6DK0 binding site are lower than those of the 6DK1 agonist binding site (−61.27 kcal/mol vs. −56.65 kcal/mol). This can be explained by the fact that the fabomotizole molecule can be stabilized inside the 6DK0 site due to five non-covalent interactions: H-bonds with Glu172 and Trp164, π-cation formation with Phe107, a salt bridge with Glu172, and CH-π interaction with Tyr103. In the agonist’s 6DK1 binding site, there are only three of them, one H-bond with Glu172, π-cation with Phe107, and a salt bridge with Glu172 (see Table 1). This does not contradict the generally accepted concept of ligand’s affinity evaluation in terms of ΔG_bind_ (energy of ‘ligand–protein’ complex formation). Along with this, the values of ΔG_bind_ calculated for NE-100 and fabomotizole in 6DK0 and (+)-pentazocine and fabomotizole in 6DK1 binding sites should be compared. Their difference (ΔΔG_bind_) is −13.49 kcal/mol (ΔG_bind NE-100_ = −74.76 kcal/mol; ΔG_bind FAB_ = −61.27 kcal/mol) in the 6DK0 case and ΔΔG_bind_ is = −3.85 kcal/mol (ΔG_bind PTZ_ is −60.50 kcal/mol; ΔG_bind FAB_ = −56.65 kcal/mol) in the case of 6DK1. Based on the comparison of these values and the coincidence of the number and type of interactions with the amino acids of the 6DK1 site (Table 1), it can be also assumed that the affinity of fabomotizole and (+)-pentazocine will also be closer than that of fabomotizole and NE-100. 

It is important to note that currently there are no definite “structure–activity” relationship criteria or specific receptor conformations allowing to distinguish between Sigma1R agonists and antagonists. This classification is based mainly on pharmacological and biochemical (functional) characterization of Sigma1R ligands. Moreover, X-ray crystal structures of Sigma1R in complex with antagonists (haloperidol, NE-100, PD144418) and agonist ((+)-pentazocine) are not significantly different, except for a 1.8 Å shift between helices α4 and α5 in the (+)-pentazocine–bound structure relative to the PD144418-bound structure [34,35]. Thus, although initial in silico data on fabomotizole/Sigma1R interaction obtained in ‘static docking approach’ is insufficient to refer to fabomotizole as agonist or antagonist, patterns of interaction mode determined for the fabomotizole and (+)-pentazocine are closer than that for fabomotizole and NE-100).

Summarizing results of our study, we can draw the following conclusions:

- Compounds with antagonistic activity to Sigma1R prevent the development of anxiolytic-like action of fabomotizole;
- Fabomotizole interaction mode with Sigma1R is similar to that observed for known Sigma1R ligands. Fabomotizole engages Glu172 residue to form an electrostatic interaction with substitute amino groups;
- Fabomotizole is located in the 6DK1 binding site of agonist (+)-pentazocine, completely reproducing the mode of its interaction with key amino acids; ΔG_bind_ values calculated for fabomotizole and (+)-pentazocine are similar (the difference is only −3.85 kcal/mol).

Therefore, despite the multitarget nature of fabomotizole, it can be concluded that Sigma1R contributes to the anxiolytic effect of fabomotizole, which is shown by the ability of Sigma1R antagonists to prevent its anxiolytic-like action which, probably, attributes to the ‘agonist-like’ interactions of fabomotizole with Sigma1R.

## 4. Materials and Methods

### 4.1. In Vivo Study

#### 4.1.1. Chemicals

The following chemicals were used: fabomotizole (ethoxy-2-[2-(morpholino)-ethylthio]benzimidazole dihydrochloride; IUPAC 4-[2-[(6-ethoxy-1*H*-benzimidazol-2-yl)sulfanyl]ethyl]morpholine dihydrochloride) (FSBI “Zakusov Institute of Pharmacology”, Moscow, Russia), BD-1047 hydrobromide (Tocris Bioscience, Bristol, UK), NE-100 hydrochloride (Santa Cruz Biotechnology, Dallas, TX, USA).

#### 4.1.2. Experimental Animals

The study was performed on male BALB/c mice (20–22 g, *n* = 99) obtained from Pushchino Breeding Center (Branch of the Institute of Bioorganic Chemistry, Russian Academy of Sciences). Animals were housed under standard vivarium conditions (20–22 °C, 30–70% humidity, 12-h light/dark cycle) in plastic cages with sawdust bedding and 6–12 animals per cage.

#### 4.1.3. Ethical Approval

All experimental procedures were approved by the bioethics committee of the FSBI “Zakusov Institute of Pharmacology”, protocol #04 of 25.02.2021. All applicable national [121] and international [122] guidelines for the care and use of experimental animals were followed.

#### 4.1.4. In Vivo Experimental Design

In vivo experimental design was developed in compliance with the 3R principles. All drug substances were dissolved in water for injections immediately before administration. Injections were made intraperitoneally (0.1 mL/10 g body weight). Fabomotizole was injected at a 2.5 mg/kg dose 30 min prior to the EPM exposition. Selective Sigma1R antagonists BD-1047 or NE-100 were injected at a 1.0 mg/kg dose 30 min prior to fabomotizole or vehicle. The animals were randomly divided as follows: intact mice (*n* = 15), mice treated with vehicle 1 and vehicle 2 (*n*  =  14), mice treated with BD-1047 and vehicle 2 (*n*  =  14), mice treated with NE-100 and vehicle 2 (*n*  =  14), mice treated with vehicle 1 and fabomotizole (*n* = 14), mice treated with BD-1047 and fabomotizole (*n* = 14), and mice treated with NE-100 and fabomotizole (*n* = 14). A power analysis was used to calculate the sample sizes.

#### 4.1.5. Elevated Plus Maze Test

The EPM (RPC OpenScience Ltd., Moscow region, Russia) was elevated 40 cm above the floor and illuminated by dim diffused light. Length and width of arms was 30 cm and 5 cm, respectively; central area was formed by a 5 × 5 cm square. Wall height of closed arms was 15 cm. The animals were kept in individual plexiglass containers after injection on the day of the experiment. For an EPM test, mice were removed from their containers and placed in the central region of the test with their head toward an open arm. The test lasted for 5 min. For each animal, time in open arms (T open), time in the center (T center), time in closed arms (T closed), number of entries into the open arms (N open), number of entries into the closed arms (N closed), and number of visits to the center (N center) were recorded. The total number of test area visits (N total) was calculated as

N total = N open + N center + N closed.(1)

Percentages of open arms visits and time spent in the open arms were calculated as

%N open = 100 × N open/(N open + N closed),(2)

%T open = 100 × T open/(T open + T closed)(3)

accordingly, abiding by recommendations [89].

#### 4.1.6. Statistical Analysis

To evaluate the experimental data distribution, D’Agostino‒Pearson and Shapiro‒Wilk tests were used. Statistical significance was calculated using two-way ANOVA with Sidak post hoc test or Kruskal‒Wallis test with Dunn’s post hoc test. Data are presented as mean with 95% CI, mean and standard deviation (mean ± S.D.), or median with 95% CI, median with lower and upper quartiles (Mdn (q25–75)). A value of *p* < 0.05 was considered to be statistically significant. Statistical analysis and visualization were performed using GraphPad Prism software version 8.0.1 for Windows (GraphPad, La Jolla, CA, USA, www.graphpad.com (accessed on 21 May 2021)).

### 4.2. Molecular Docking

#### 4.2.1. Set of Compounds and RCSB Protein Data Bank Codes

The reported crystal structures of Sigma1R with corresponding ligands (PDB IDs: 6DK0, 6DK1 https://www.rcsb.org (accessed on 21 May 2021)) [34,35] and the A chain of the Sigma1R were used for comparative docking studies of fabomotizole, NE-100, and (+)-pentazocine. Data on their K_i_ values were obtained from the references [34,123] and our own experiments [80] (Table S3).

#### 4.2.2. Ligand Preparation

All following calculations were performed in demo version of Schrödinger Suites 2021-1 program complex (Schrödinger Release 2021-1: Maestro, Schrödinger, LLC, New York, NY, USA, 2021) permitted on March 03.2021 for FSBI “Zakusov Institute of Pharmacology”, Moscow, Russia. The ligand structures were sketched using ‘Maestro’ molecular editor. Those structures were prepared for subsequent procedures via ‘LigPrep’ module: geometries were optimized using OPLS4e [124] force field, and their ionization states were generated at pH 7.0 ± 2.0 using ‘Epik’ [125]. To evaluate additional properties of the binding site conformations of the aforementioned structures the ‘SiteMap’ module was used.

#### 4.2.3. Protein Preparation

Following additional procedures were carried out with the respective receptor structure (PDB IDs: 6DK0, 6DK1) using the ‘Protein Preparation Wizard’ module [126]: unspecified side chains and loops were restored using the ‘Prime’ utility [127,128]. After these modifications, the search for hydrogen bonds was performed and hydrogen bonds were re-declared. Co-crystallized ligands were deleted from PDB structures. Water and other irrelevant molecules were removed, and restrained minimization of the geometric structure of the complexes was performed. A number of receptor grids was generated to define a ligand binding site for subsequent docking analysis. A grid box of 20 × 20 × 20 Å was created for each ‘ligand–receptor’ complex, centered on the center of mass of the ligand in the selected crystal structure, covering the binding site of Sigma1R [86]. A scaling factor of 1.0 and a partial charge threshold of 0.25 were used during the generation of the grid boxes. Additionally, all possible hydroxyl and thiol groups in the vicinity of the active center of the receptor that could undergo rotation were marked as rotatable.

#### 4.2.4. Docking Protocols and Calculations

The generated grid boxes (for each of PDB structure) were then used for re-docking of reference ligands and docking of fabomotizole using the Glide extra-precision protocol [129,130,131] at the first step. The best poses obtained during this procedure then were used for following MM_GBSA calculations, in order to obtain the ΔG_bind_ values. To calculate the binding energy between ligand and the Sigma1R in the ‘ligand–protein complex’ (solvent—water, the flexibility of the protein is limited within a 3 Å radius from the ligand), the generalized Born model (GB) was used, taking into account the available surface area (SA) in the context of molecular mechanics (MM): ΔG_bind_ (MM_GBSA).

#### 4.2.5. Docking Study Design

The study comprised the following steps:

1. Docking (Glide XP + MM_GBSA) of fabomotizole into the structures of Sigma1R bound to (+)-pentazocine (PDB ID: 6DK1) and bound to NE-100 (PDB ID: 6DK0) with comparison of calculated parameters;
2. Calculation of the binding energies (ΔG_bind_) for the best binding poses of fabomotizole and reference ligands, using Glide XP + MM_GBSA approach; calculation of ΔΔG_bind_ values as a difference between ΔG_bind_/ref and ΔG_bind_/fab—one of the major characteristics for evaluation of similarity of fabomotizole to one of the tested references Sigma1R ligands;
3. Comparisons of the pattern of interactions with the amino acids of the Sigma1R binding site and location inside of Sigma1R binding site and binding mode between fabomotizole and reference ligands (analysis of hydrogen bond interactions, π-π stacking interaction, π-cation, and salt bridge formations).

## Figures and Tables

**Table 1:** Results of molecular docking procedure.

PDB ID	Ligand	Glide XP + MM_GBSA Mode				RMSD, Å
		Residues in H-Bond Interaction	Other Interactions	ΔG_bind_, kcal/mol	ΔΔG_bind,_ kcal/mol	
6DK0	NE-100	-	salt bridge: Glu172 π-cation: Phe107	−74.76	−13.49	1.032
	fabomotizole	Glu172, Trp164	π-cation: Phe107 salt bridge: Glu172 CH-π: Tyr103	−61.27		-
6DK1	(+)-pentazocine	Glu172	π -cation: Phe107 salt bridge: Glu172	−60.50	−3.85	0.301
	fabomotizole	Glu172	π -cation: Phe107 salt bridge: Glu172	−56.65		-

## Data Availability

The data presented in this study are openly available in www.synapse.org: https://www.synapse.org/#!Synapse:syn25328722 (accessed on 21 May 2021). The data presented in this study are also available in Table S3.

## Supplementary Materials

The following are available online at https://www.mdpi.com/article/10.3390/ijms22115455/s1, Figure S1: The influence of Sigma1R antagonists on the effect of anxiolytic dose of fabomotizole on the closed arm entries and the total entries of the elevated plus-maze, Figure S2: Superposition of 6DK0 (green) and 6DK1 (purple) active sites, Figure S3: Superposition of 4α-helix of 6DK0 (green) and 4α-helix of 6DK1 (purple) from ILE178 to GLN194 (left to right), Figure S4a: The distances between the core carbons of the selected AAs that belonging α4-helix (blue) and α5-helix (violet) of the 6DK0 binding site with NE-100 inside, Figure S4b: The distances between the core carbons of the selected AAs that belonging α4-helix (blue) and α5-helix (violet) of the 6DK1 binding site with PTZ inside, Figure S5a: Docking of NE-100 in 6DK0 binding site (ΔG_bind_ = −74.76 kcal/mol), Figure S5b: Docking of NE-100 in 6DK1 binding site (ΔG_bind_ = −69.29 kcal/mol), Figure S6a: Docking of PTZ in 6DK0 binding site (ΔG_bind_ = −34.59 kcal/mol), Figure S6b: Docking of PTZ in 6DK1 binding site (ΔG_bind_ = −60.50 kcal/mol), Figure S7a: Docking of FAB in 6DK0 binding site (ΔG_bind_ = −61.27 kcal/mol), Figure S7b: Docking of FAB in 6DK1 binding site (ΔG_bind_ = −56.65 kcal/mol), Figure S8: Results of the ‘SiteMap’ assay for the 6DK0 binding site, Figure S9: Results of the ‘SiteMap’ assay for the 6DK1 binding site, Table S1: The influence of Sigma1R antagonists on the effect of anxiolytic dose of fabomotizole on the entries and the time spent in open arms of the elevated plus-maze test, Table S2: The influence of Sigma1R antagonists on the effect of anxiolytic dose of fabomotizole on the entries into closed arms and the total entries of the elevated plus-maze, Table S3: Structures of the studied Sigma1R ligands and their experimental K_i_ values, Table S4: Values of ΔG_bind_ (MM/GBSA) calculated for NE-100, (+)-pentazocine and fabomotizole as the results of docking into 6DK0 and 6DK1 binding sites.

## Abbreviations

4-IBP	N-(1-benzylpiperidin-4-yl)-4-iodobenzamide
5-HT	5-hydroxytryptamine; serotonin
ASIC	Acid-sensing ion channel
BD-1047	*N*′-[2-(3,4-dichlorophenyl)ethyl]-*N*,*N*,*N*′-trimethylethane-1,2-diamine
BD-1063	1-[2-(3,4-dichlorophenyl)ethyl]-4-methylpiperazine
BDNF	Brain-derived neurotrophic factor; human BDNF gene
BiP	Endoplasmic reticulum chaperone BiP
BMY-14802	1-(4-fluorophenyl)-4-[4-(5-fluoropyrimidin-2-yl)piperazin-1-yl]butan-1-ol
CB_1_R	Cannabinoid receptor 1
*Cnr1*	Rodent cannabinoid receptor 1 gene
D_1_R	D(1A) dopamine receptor
D_2_R	D(2) dopamine receptor
DAT	Sodium-dependent dopamine transporter
ΔG_bind_	The binding free energy
DHEA	Dehydroepiandrosterone
DHEAS	Dehydroepiandrosterone sulfate
EPM	Elevated plus maze test
ER	Endoplasmic reticulum
FAB	Fabomotizole; 4-[2-[(6-ethoxy-1*H*-benzimidazol-2-yl)sulfanyl]ethyl]morpholine dihydrochloride
GABA	gamma-aminobutyric acid type; 4-aminobutanoic acid
GluN	Glutamate receptor ionotropic, NMDA
HINT1	Histidine triad nucleotide-binding protein 1
IRE1	Serine/threonine-protein kinase/endoribonuclease IRE1
JO-1784	(E)-N-(cyclopropylmethyl)-N-methyl-3,6-diphenylhex-5-en-3-amine hydrochloride
MAM	Mitochondria-associated membranes
MAO-A	Amine oxidase [flavin-containing] A
MT_3_ receptor	Melatonin receptor, type 3
NE-100	*N*-[2-[4-methoxy-3-(2-phenylethoxy)phenyl]ethyl]-*N*-propylpropan-1-amine
nH	Hill coefficient
NMDA	(2R)-2-(methylamino)butanedioic acid
NQO2	NRH:quinone oxidoreductase 2; ribosyldihydronicotinamide dehydrogenase [quinone]
NPY	Pro-neuropeptide Y
NRH	dihydronicotinamide riboside;
	1-[(2*R*,3*R*,4*S*,5*R*)-3,4-dihydroxy-5-(hydroxymethyl)oxolan-2-yl]-4*H*-pyridine-3-carboxamide
OF	Open field test
PTZ	(+)-pentazocine; (1*S*,9*S*,13*S*)-1,13-dimethyl-10-(3-methylbut-2-enyl)-10-azatricyclo[7.3.1.0^2,7^]trideca-2(7),3,5-trien-4-ol
PCP	phencyclidine; 1-(1-phenylcyclohexyl)piperidine
PD144418	3-(4-methylphenyl)-5-(1-propyl-3,6-dihydro-2H-pyridin-5-yl)-1,2-oxazole
PDB	Protein Data Bank
PRE-084	2-morpholin-4-ylethyl 1-phenylcyclohexane-1-carboxylate
PREGS	Pregnenolone sulfate
PROG	Progesterone
PTSD	Post-traumatic stress disorder
RCSB	Research Collaboratory for Structural Bioinformatics
Sigma1R	Sigma nonopioid intracellular receptor 1; chaperon Sigma1R
*Sigmar1*	Rodent Sigma1R gene
(+)-SKF 10.047	(1*S*,9*S*,13*S*)-1,13-dimethyl-10-prop-2-enyl-10-azatricyclo[7.3.1.0^2,7^]trideca-2(7),3,5-trien-4-ol
RMSD	Root-mean-square deviation
SNRI	Serotonin-noradrenaline reuptake inhibitor
SSRI	Selective serotonin reuptake inhibitor
UPR	Unfolded protein response
XBP1	X-box-binding protein 1
