# Supplementary material for: Involvement of Chaperone Sigma1R in the Anxiolytic Effect of Fabomotizole
Source: Int J Mol Sci. 2021 May 21;22(11):5455. doi: 10.3390/ijms22115455 (PMC8196847; doi:10.3390/ijms22115455)
Supplement: Supplementary file 1 [file ijms-22-05455-s001.zip › ijms-1184251-supplementary-proof.pdf]

International Journal of
*Molecular Sciences*

**Supplementary Table S1.** The influence of Sigma1R antagonists on the effect of anxiolytic dose of fabomotizole on the entries and the time spent in open arms of the elevated plus-maze test.

| Experimental Groups | Number of Entries into Open Arms (N Open) | Number of Entries into Open Arms, % (%N Open) | Time Spent in Open Arms, s (T Open) | Time Spent in Open Arms, % (%T Open) |
|---|---|---|---|---|
| Intact<br>*n* = 15 | 0.0 (0.0–0.0) | 0.0 (0.0–0.0) | 0.0 (0.0–0.0) | 0.0 (0.0–0.0) |
| Veh1 + Veh2<br>*n* = 14 | 0.0 (0.0–0.0) | 0.0 (0.0–0.0) | 0.0 (0.0–0.0) | 0.0 (0.0–0.0) |
| BD-1047 1.0 + Veh2<br>*n* = 14 | 0.0 (0.0–1.0) | 0.0 (0.0–13.0) | 0.0 (0.0–2.0) | 0.0 (0.0–0.7) |
| NE-100 1.0 + Veh2<br>*n* = 14 | 1.0 (0.75–1.25)<br>*$p$ = 0,032 | 10.5 (6.83–25.50) | 3.5 (0.75–9.0)<br>*$p$ = 0,048 | 1.6 (0.38–3.83)<br>*$p$ = 0,0411 |
| Veh1 + Fab2.5<br>*n* = 14 | 4.0 (3.0–5.0)<br>*$p < 0.001$ | 47.0 (32.50–57.75)<br>*$p < 0.0001$ | 52.5 (31.5–102.5)<br>*$p < 0.001$ | 35.0 (13.73–64.0)<br>*$p < 0.001$ |
| BD-1047 1.0 + Fab2.5<br>*n* = 14 | 0.0 (0.0–0.0)<br>#$p < 0.001$ | 0.0 (0.0–0.0)<br>#$p < 0.001$ | 0.0 (0.0–0.0)<br>#$p < 0.001$ | 0.0 (0.0–0.0)<br>#$p < 0.001$ |
| NE-100 1.0 + Fab2.5<br>*n* = 14 | 0.5 (0.0–3.0)<br>#$p = 0.005$ | 5.5 (0.0–25.5)<br>#$p = 0.002$ | 1.5 (0.0–13.0)<br>#$p = 0.001$ | 0.55 (0.0–5.9)<br>#$p = 0.001$ |

Data are presented as median (q25–75). *n*–the number of animals in the experimental group. Experimental groups: intact BALB/c mice (Intact), vehicle 1 + vehicle 2 (Veh1 + Veh2), BD-1047 1.0 mg/kg + vehicle 2 (BD-1047 1.0 + Veh2), NE-100 1.0 mg/kg + vehicle 2 (NE-100 1.0 + Veh2), vehicle 1 + fabomotizole 2.5 mg/kg (Veh1 + Fab 2.5), BD-1047 1.0 mg/kg + fabomotizole 2.5 mg/kg (BD-1047 1.0 + Fab 2.5), NE-100 1.0 mg/kg + fabomotizole 2.5 mg/kg (NE-100 1.0 + Fab 2.5). Kruskal–Wallis test, Dunn's multiple comparison test: *: statistical significance vs. Veh1 + Veh2, #: statistical significance vs. Veh1 + Fab2.5.

**Supplementary Table S2.** The influence of Sigma1R antagonists on the effect of anxiolytic dose of fabomotizole on the entries into closed arms and the total entries of the elevated plus-maze.

| Experimental Groups | Number of Entries into Closed Arms (N Closed) | Number of Total Entries (N Total) |
|---|---|---|
| Intact<br>$n = 15$ | 5.87 ± 2.03 | 12.07 ± 4.18 |
| Veh1 + Veh2<br>$n$=14 | 5.93 ± 1.33 | 12.36 ± 2.87 |
| BD-1047 1.0 + Veh2<br>$n = 14$ | 6.50 ± 1.87 | 13.93 ± 3.54 |
| NE-100 1.0 + Veh2<br>$n = 14$ | 7.21 ± 2.08 | 16.93 ± 4.55 |
| Veh1+Fab2.5<br>$n = 14$ | 4.57 ± 2.21 | 17.57 ± 6.06<br>$^{*}p$ = 0,045 |
| BD-1047 1.0 + Fab2.5<br>$n = 14$ | 3.00 ± 1.30<br>$^{*}p$ = 0,002<br>$^{b}p = 0.001$ | 6.71 ± 2.59<br>$^{*}p$ = 0,022<br>$^{\#}p < 0.001$<br>$^{b}p = 0.001$ |
| NE-100 1.0 + Fab2.5<br>$n = 14$ | 7.29 ± 2.76<br>$^{\#}p = 0.006$<br>$^{\wedge}p < 0.001$ | 17.43 ± 7.01<br>$^{\wedge}p < 0.001$ |

Data are presented as mean ± S.D. $n$-the number of animals in the experimental group. Experimental groups: intact BALB/c mice (Intact), vehicle 1 + vehicle 2 (Veh1 + Veh2), BD-1047 1.0 mg/kg + vehicle 2 (BD-1047 1.0 + Veh2), NE-100 1.0 mg/kg + vehicle 2 (NE-100 1.0 + Veh2), vehicle 1 + fabomotizole 2.5 mg/kg (Veh1 + Fab 2.5), BD-1047 1.0 mg/kg + fabomotizole 2.5 mg/kg (BD-1047 1.0 + Fab 2.5), NE-100 1.0 mg/kg + fabomotizole 2.5 mg/kg (NE-100 1.0 + Fab 2.5). One-way ANOVA and the post hoc Sidak multiple comparisons test: *: statistical significance vs. Veh1 + Veh2, #: statistical significance vs. Veh1 + Fab2.5, b: statistical significance vs. BD-1047 1.0 + Veh2, ^: statistical significance vs. BD-1047 1.0 + Fab2.5.

**Supplementary Table S3.** Structures of the studied Sigma1R ligands and their experimental $K_i$ values.

| Ligands | Chemical Structure | $K_{i\ experimental}$, M | $pK_{i\ experimental}$ |
|---|---|---|---|
| NE-100 | 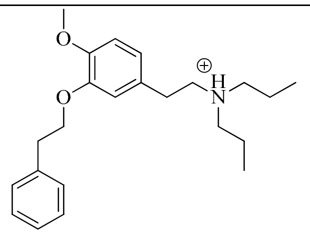 | $1.03 \times 10^{-9}$<br>[1] | 8.987 |
| (+)-pentazocine (PTZ) | 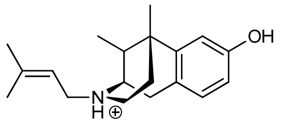 | $1.91 \times 10^{-9}$<br>[1] | 8.719 |
| fabomotizole (FAB) | 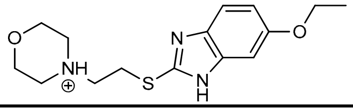 | $5.9 \times 10^{-6}$<br>[2] | 5.229 |

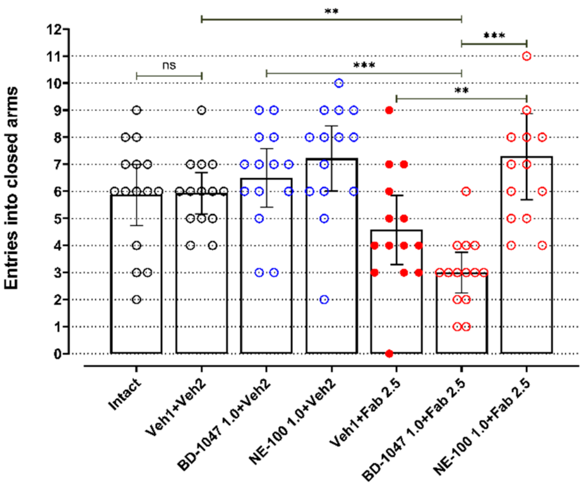


(**a**)

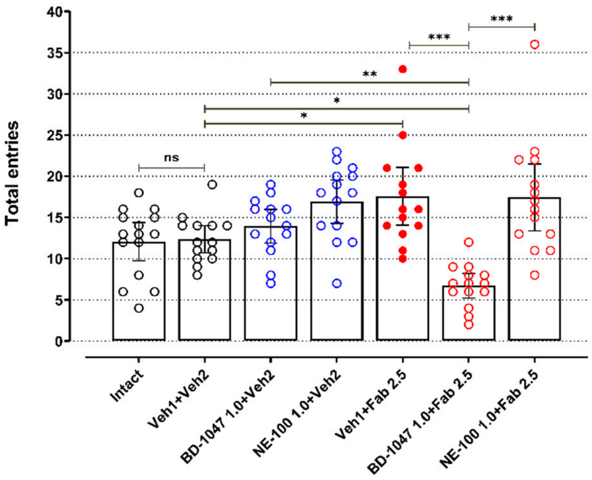


(**b**)

**Supplementary Figure S1.** The influence of Sigma1R antagonists on the effect of anxiolytic dose of fabomotizole on the closed arm entries and the total entries of the elevated plus-maze. (**a**) The number of entries into the closed arms (N closed); (**b**) The number of total entries into the open and closed arms (N total). Experimental groups were divided by drug administration: intact BALB/c mice (Intact), vehicle 1 + vehicle 2 (Veh1 + Veh2), BD-1047 1.0 mg/kg + vehicle 2 (BD-1047 1.0 + Veh2), NE-100 1.0 mg/kg + vehicle 2 (NE-100 1.0 + Veh2), vehicle 1 + fabomotizole 2.5 mg/kg (Veh1 + Fab 2.5), BD-1047 1.0 mg/kg + fabomotizole 2.5 mg/kg (BD-1047 1.0 + Fab 2.5), NE-100 1.0 mg/kg + fabomotizole 2.5 mg/kg (NE-100 1.0 + Fab 2.5). Data are presented as mean with 95% CI. Statistically significant differences according to the one-way ANOVA and the post hoc Sidak multiple comparisons test: ns - not significant; *$p$ < 0,05; **$p$ < 0,01; ***$p$ < 0,001.

Figure **S2** shows, that this difference is sufficient to attract attention; it was found in MET93, SER117, TYR120, PHE133. The distance between identical atoms in 6DK0 and 6DK1 structures for the side functional groups of these AAs are ranged from 0.76 Å to 1.72 Å. Relative to 6DK0, in 6DK1, noted functional groups are displaced in space 'downwards towards the membrane' against the direction of $\alpha$1-helix, towards the $\alpha$4-helix (figure **S2**). The deviation in the geometry of the core atoms of the $\alpha$4-helix in the 6DK0 and 6DK1 geometries presented in figure **S3**. This deviation in the position of the core atoms was measured for the residues of 178–194 AAs: it ranged from 0.27 Å to 1.51 Å.

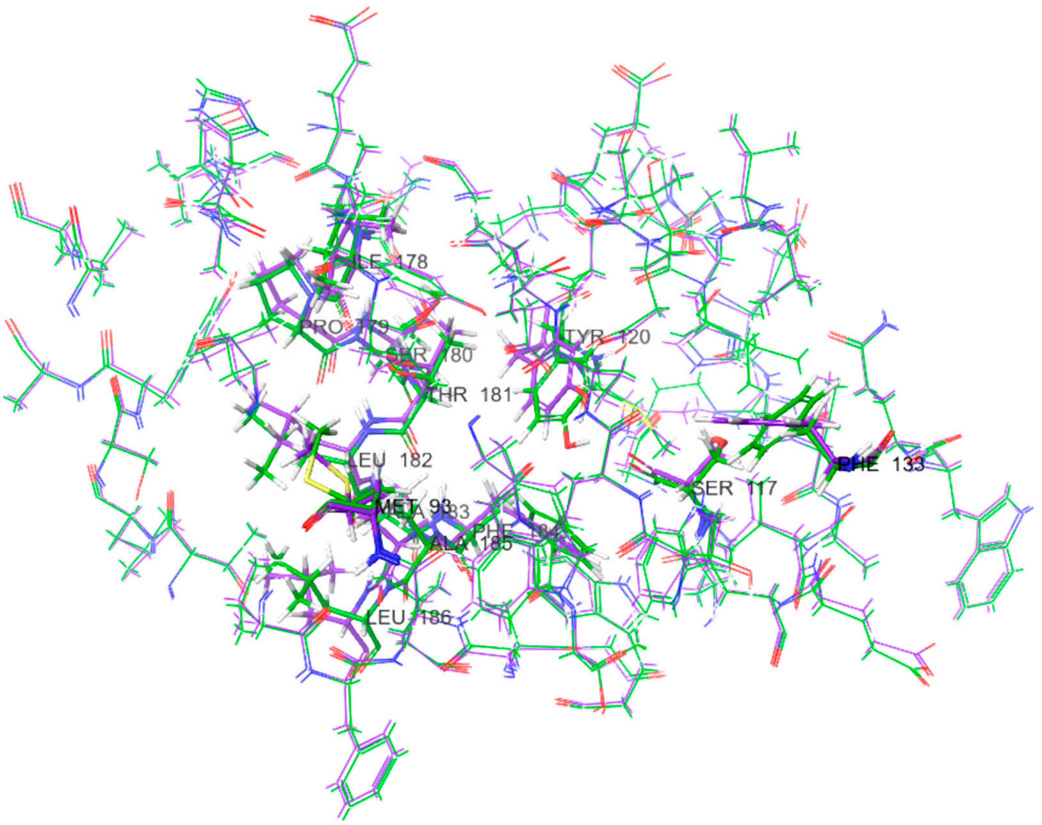


**Supplementary Figure S2.** Superposition of 6DK0 (green) and 6DK1 (purple) active sites.

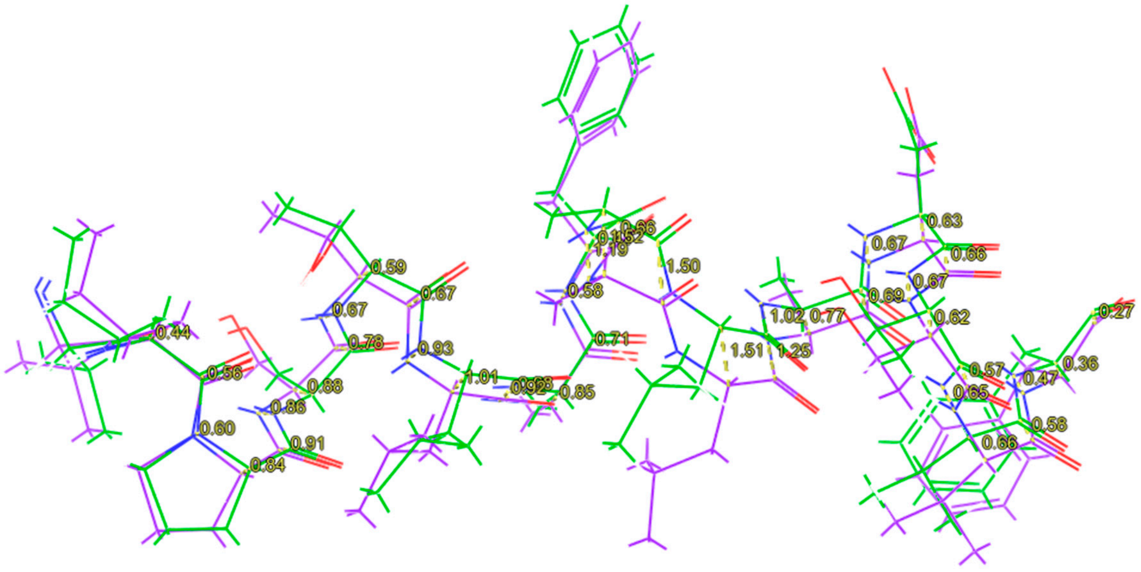

**Supplementary Figure S3.** Superposition of 4α-helix of 6DK0 (green) and α4-helix of 6DK1 (purple) from ILE178 to GLN194 (left to right).

The distances between the core carbon atoms of the selected AAs included in the α4-helix and α5-helix for both 6DK0 and 6DK1 binding site geometries are presented in figures **S4a** and **S4b**.

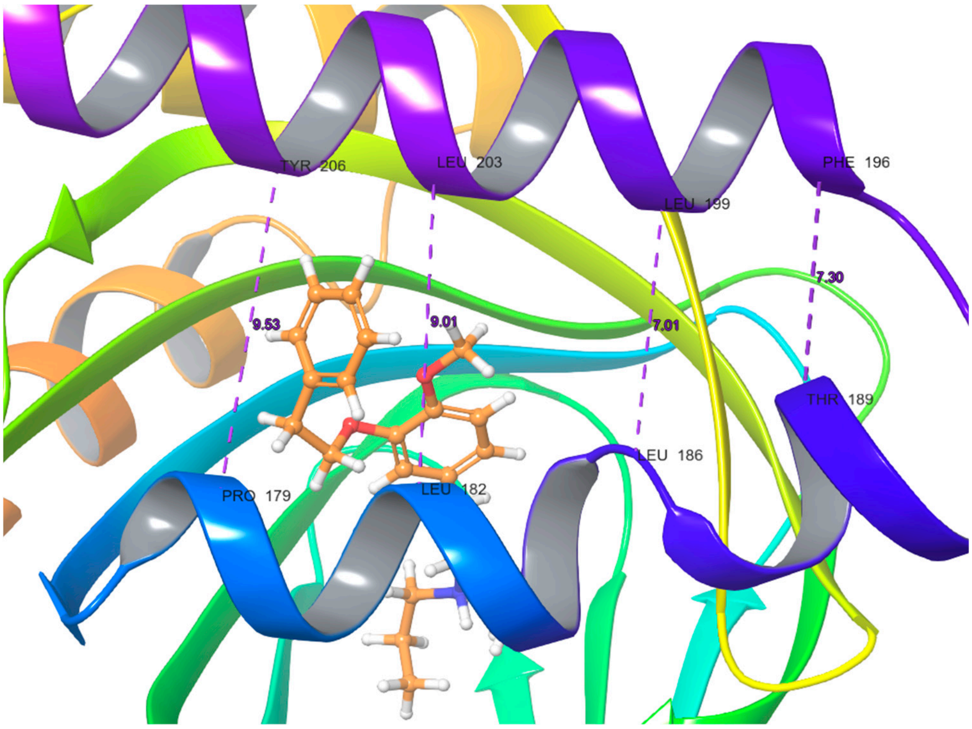


**Supplementary Figure S4a**. The distances between the core carbons of the selected AAs that belonging $\alpha$4-helix (blue) and $\alpha$5-helix (violet) of the 6DK0 binding site with NE-100 inside.

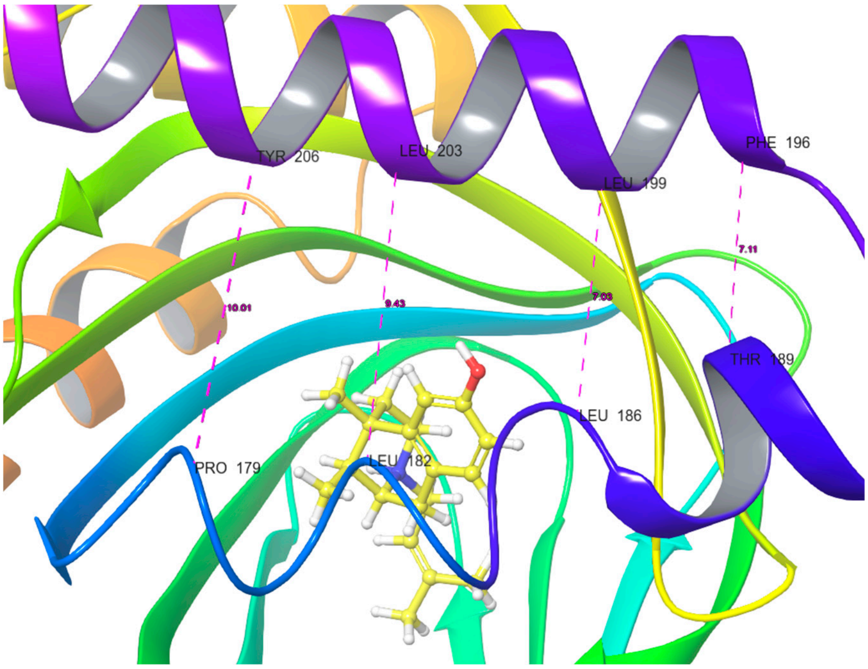


**Supplementary Figure S4b**. The distances between the core carbons of the selected AAs that belonging $\alpha$4-helix (blue) and $\alpha$5-helix (violet) of the 6DK1 binding site with PTZ inside Results of NE-100 and PTZ cross-docking procedures.

A comparison of the positions of the NE-100 and PTZ ligands in their "native" and "adjacent" binding sites and difference of conformations are presented in Figures **S5**, **S6**.

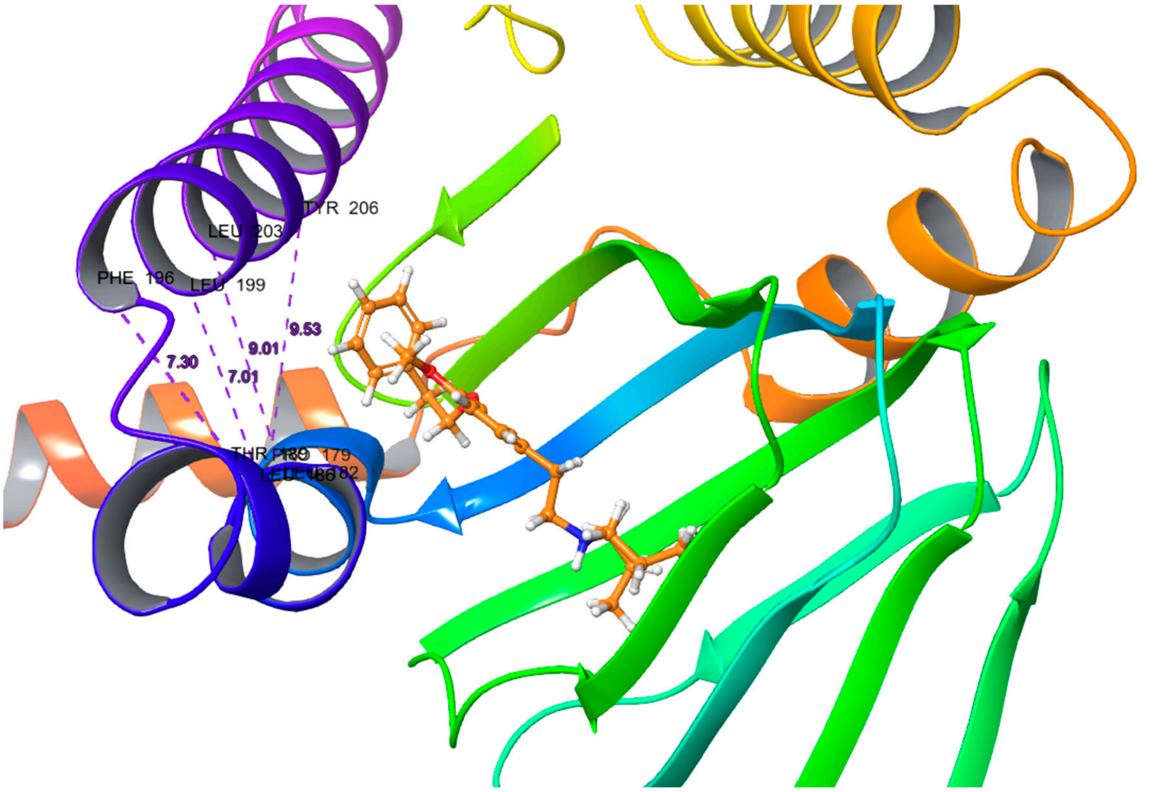


**Supplementary Figure S5a**. Docking of NE-100 in 6DK0 binding site ($\Delta G_{bind}$ = −74.76 kcal/mol).

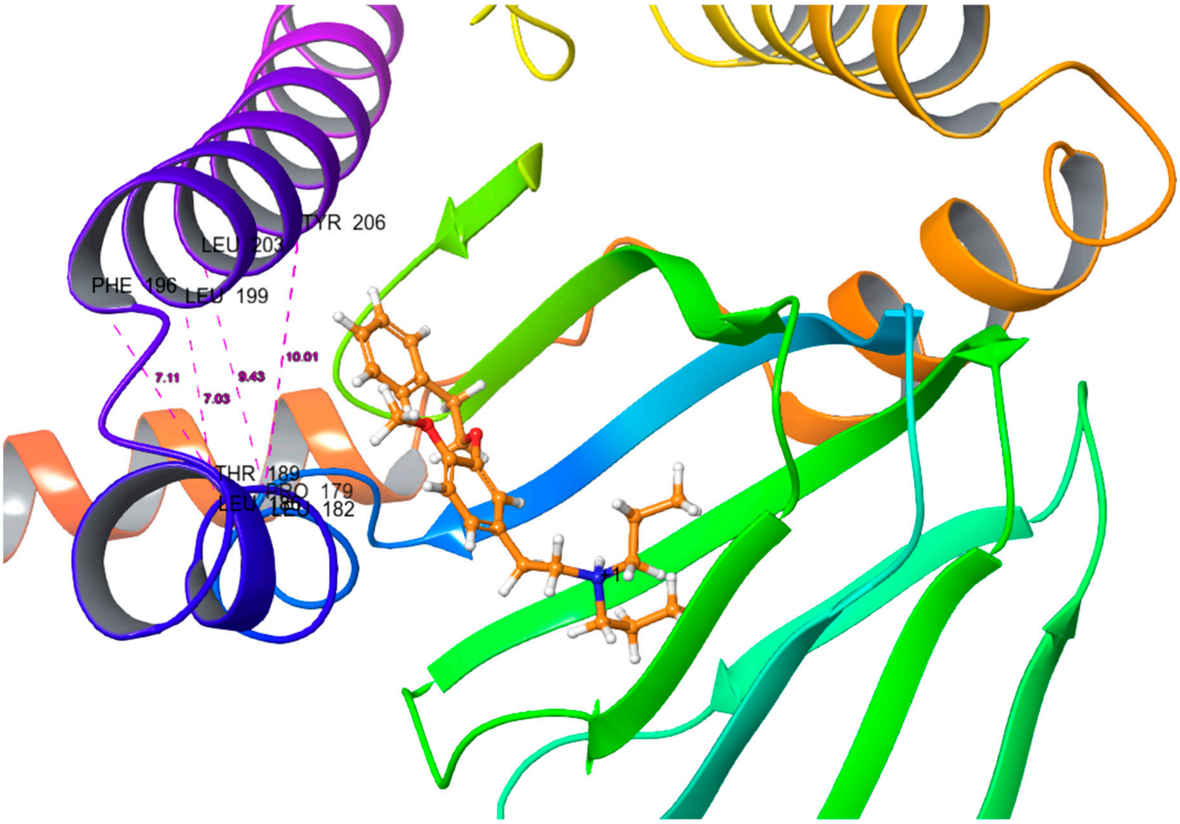


**Supplementary Figure S5b**. Docking of NE-100 in 6DK1 binding site ($\Delta G_{bind}$ = −69.29 kcal/mol).

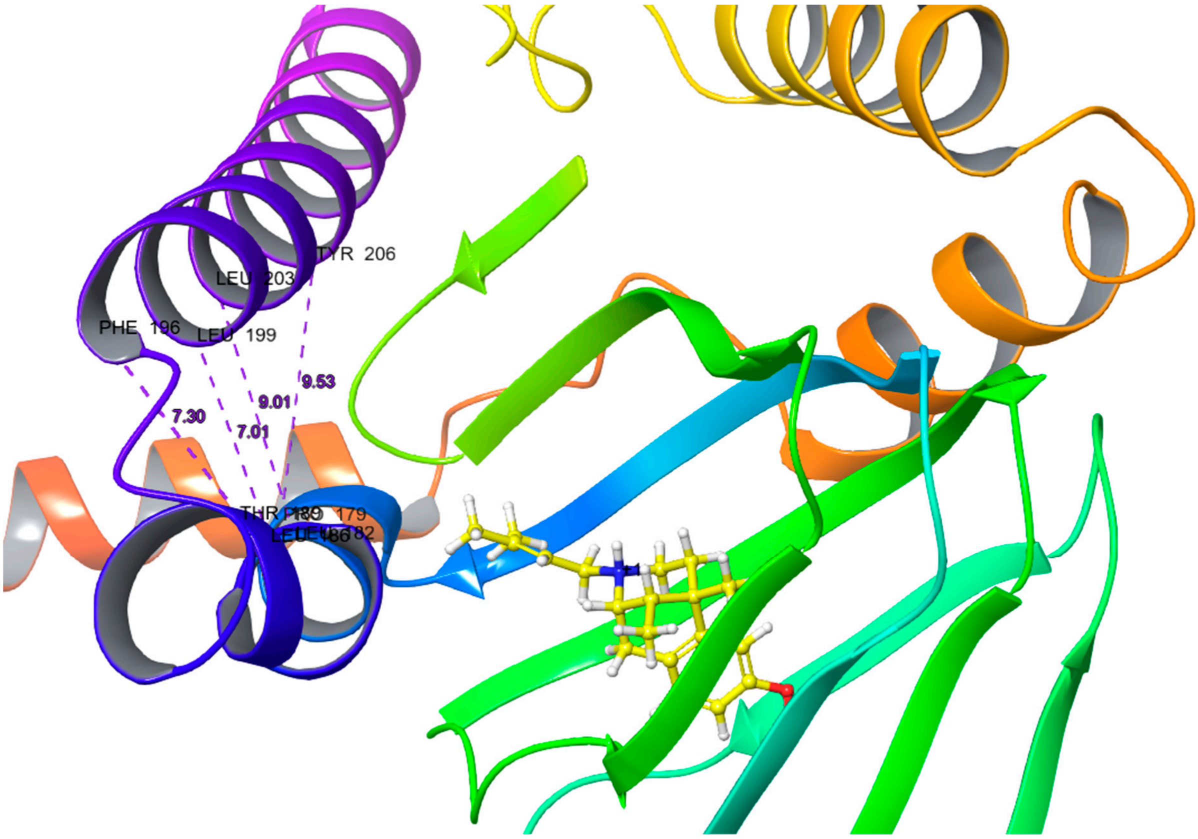


**Supplementary Figure S6a**. Docking of PTZ in 6DK0 binding site (ΔGbind = –34.59 kcal/mol).

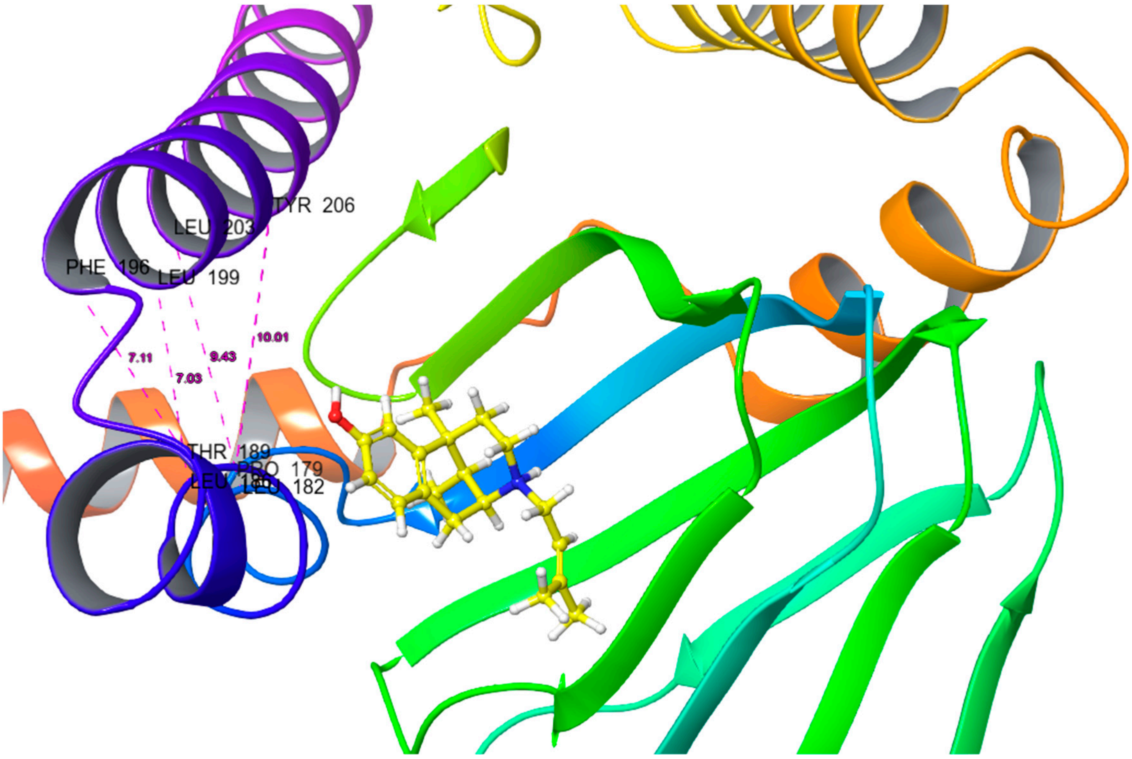


**Supplementary Figure S6b**. Docking of PTZ in 6DK1 binding site (ΔGbind = –60.50 kcal/mol).

Docking of fabomotizole produces a similar result (Figure **S7**)

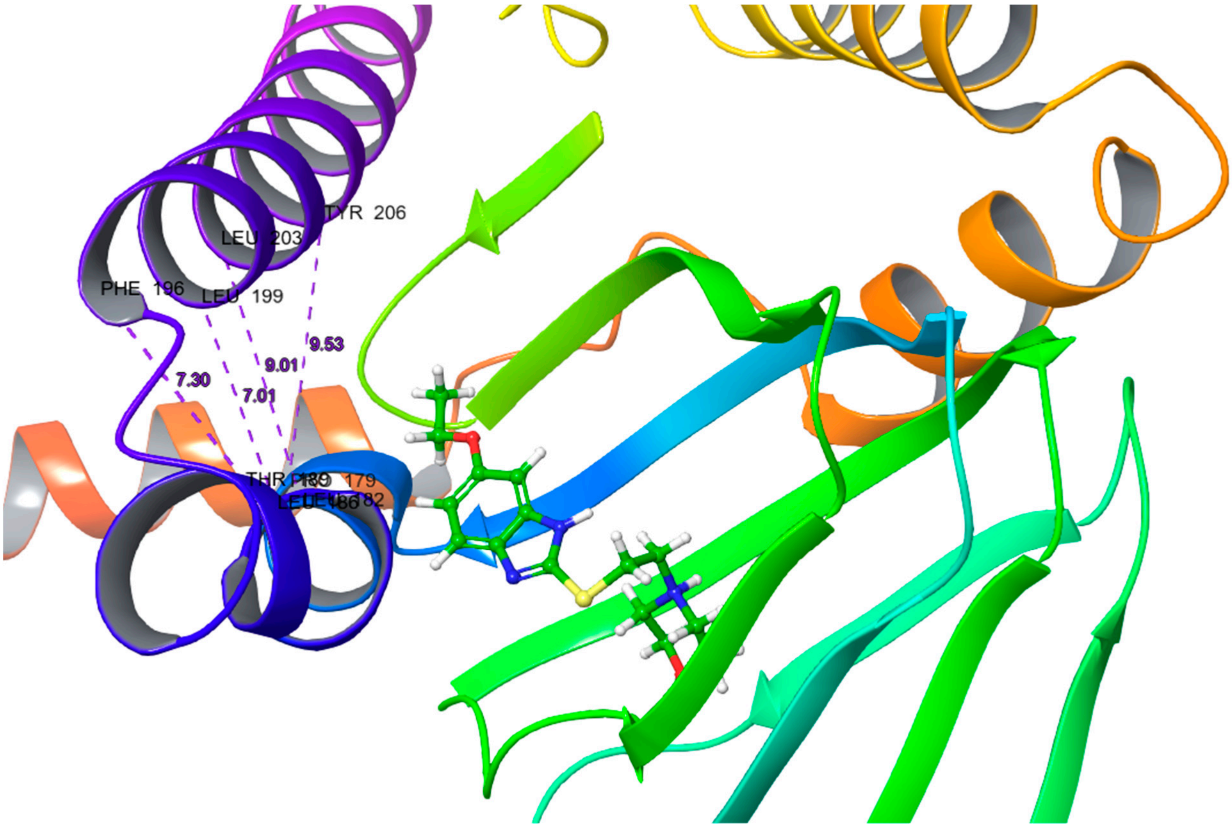


**Supplementary Figure S7a**. Docking of FAB in 6DK0 binding site ($\Delta G_{bind}$ = –61.27 kcal/mol).

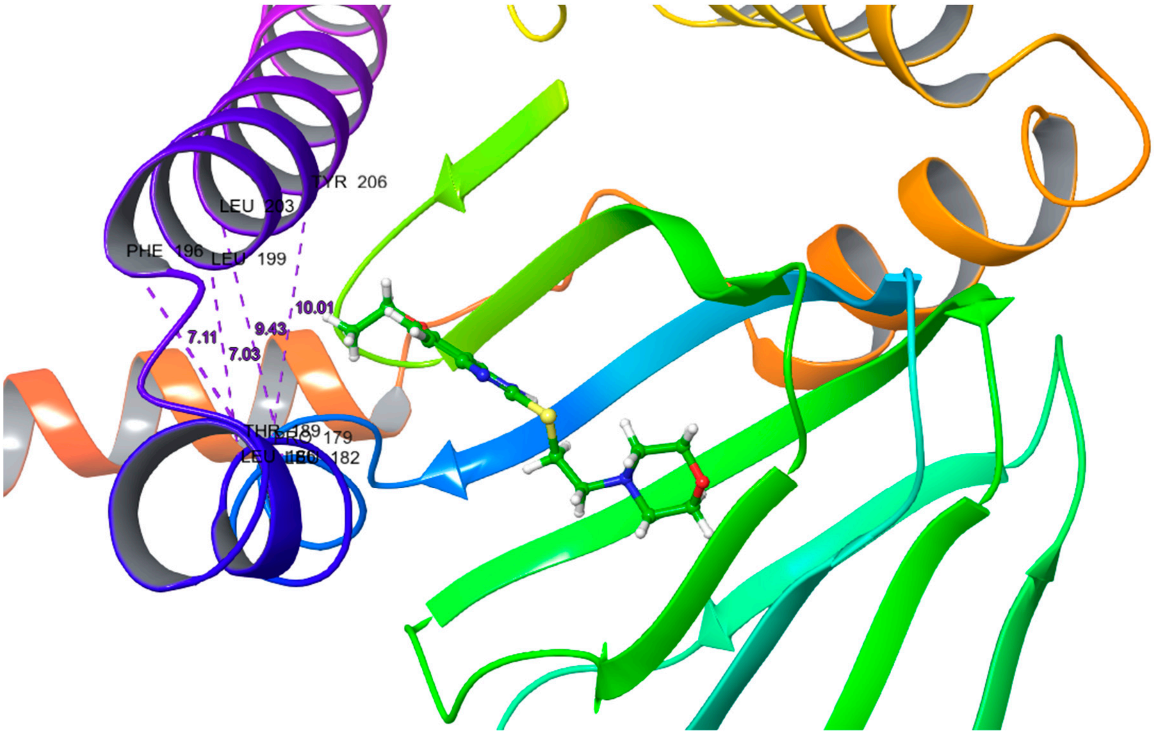


**Supplementary Figure S7b**. Docking of FAB in 6DK1 binding site ($\Delta G_{bind}$ = –56.65 kcal/mol).

**Binding energy values-ΔG$_{bind}$**

**Supplementary Table S4.** Values of ΔG$_{bind}$ (MM/GBSA) calculated for NE-100, (+)-pentazocine and fabomotizole as the results of docking into 6DK0 and 6DK1 binding sites.

| Entry | Ligand | ΔG$_{bind}$ (6DK0) | ΔG$_{bind}$ (6DK1) |
|---|---|---|---|
| 1 | NE-100 | −74.76 kcal/mol | −69.29 kcal/mol |
| 2 | (+)-pentazocine (PTZ) | −34.59 kcal/mol | −60.50 kcal/mol |
| 3 | fabomotizole (FAB) | −61.27 kcal/mol | −56.65 kcal/mol |

**The results of the 'SiteMap' assay of 6DK0 and 6DK1 active site conformations**

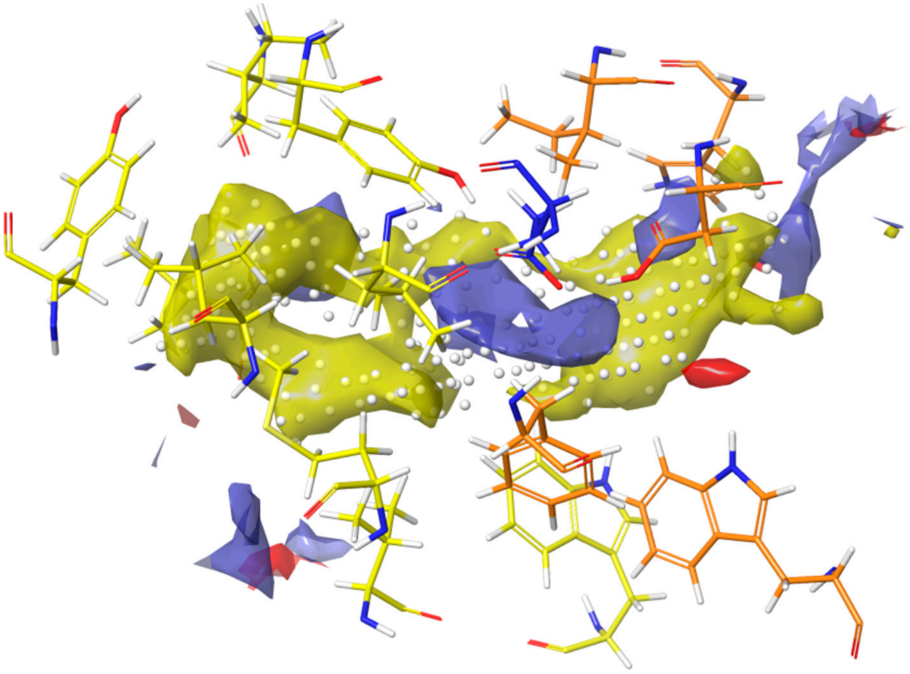

**Supplementary Figure S8.** Results of the 'SiteMap' assay for the **6DK0** binding site. Glu172 carboxylate is represented in blue color, amino acids of primary hydrophobic site (Val84, Trp89, Met93, Leu95, Leu105, Tyr206, Ile178, Leu182, Tyr103) are represented in yellow color, and residues of secondary hydrophobic site (Phe107, Trp164, His154, Ile124, Asp126) are represented in orange. White dots represent the internal method of evaluating and mapping available volume; hydrophobic regions are mapped in yellow, H-bond donor regions are mapped in blue, and H-bond acceptors are mapped in red.

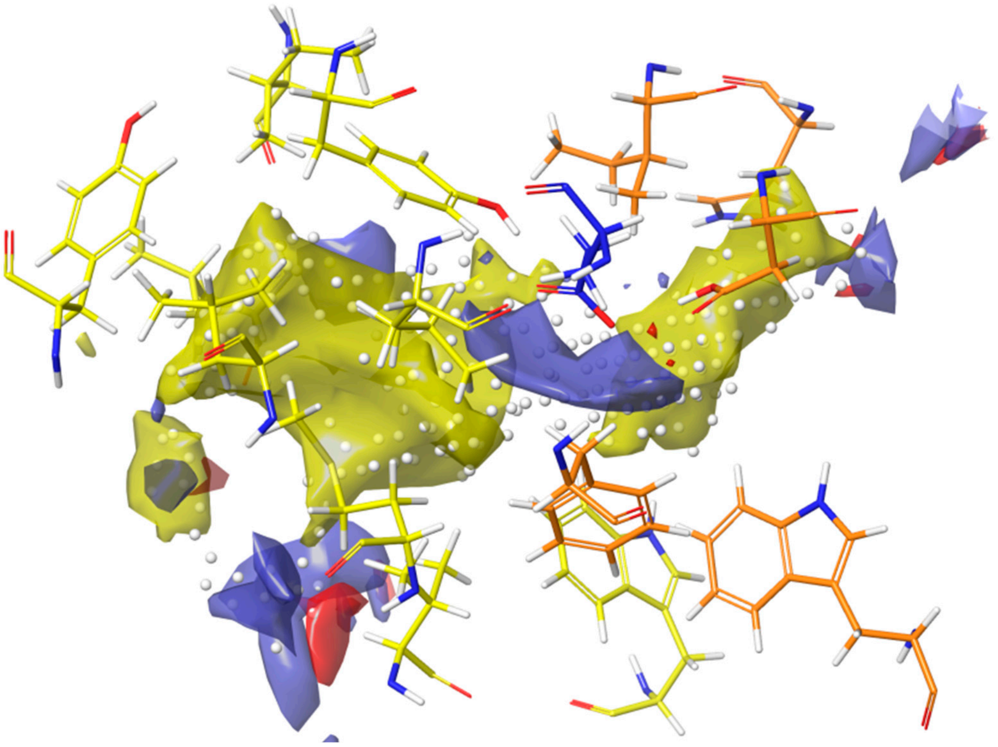

**Supplementary Figure S9.** Results of the 'SiteMap' assay for the **6DK1** binding site. Glu172 carboxylate is represented in blue color, amino acids of primary hydrophobic site (Val84, Trp89, Met93, Leu95, Leu105, Tyr206, Ile178, Leu182, Tyr103) are represented in yellow color, and residues of secondary hydrophobic site (Phe107, Trp164, His154, Ile124, Asp126) are represented in orange. White dots represent the internal method of evaluating and mapping available volume; hydrophobic regions are mapped in yellow, H-bond donor regions are mapped in blue, and H-bond acceptors are mapped in red.

## References

1. Berardi, F.; Ferorelli, S.; Colabufo, N.A.; Leopoldo, M.; Perrone, R.; Tortorella, V. A multireceptorial binding reinvestigation on an extended class of sigma ligands: N-[omega-(indan-1-yl and tetralin-1-yl)alkyl] derivatives of 3,3-dimethylpiperidine reveal high affinities towards sigma1 and EBP sites. *Bioorg Med Chem* **2001**, *9*, 1325–1335, doi:10.1016/s0968-0896(01)00011-6.
2. Seredenin, S.B.; Antipova, T.A.; Voronin, M.V.; Kurchashova, S.Y.; Kuimov, A.N. Interaction of afobazole with sigma1-receptors. *Bull Exp Biol Med* **2009**, *148*, 42–44, doi:10.1007/s10517-009-0624-x.
